# A CSF-1R inhibitor both prevents and treats triple-negative breast cancer brain metastases in hematogenous preclinical models

**DOI:** 10.1007/s10585-025-10366-x

**Published:** 2025-08-04

**Authors:** Wei Zhang, Samiur Rahman, Alex M. L. Wu, Kristine Isanogle, Christina Robinson, Dinesh Kumar, Imran Khan, Debbie Wei, Alexandra S. Zimmer, Takeo Fujii, Simone Difilippantonio, Stanley Lipkowitz, Patricia Steeg

**Affiliations:** 1 Women’s Malignancies Branch, Center for Cancer Research, National Cancer Institute, Building 37, Room 1126, Bethesda, MD 20892 USA; 2 Animal Research Technical Support, Frederick National Laboratory for Cancer Research, National Cancer Institute, Frederick, MD USA

**Keywords:** Brain metastasis, Triple-negative breast cancer, Colony-stimulating factor-1, Colony-stimulating factor-1 receptor, BLZ945, Microglia, Astrocyte, Macrophage, Tumor microenvironment

## Abstract

Brain metastasis is a common and serious complication of metastatic triple-negative breast cancer (TNBC) with few effective treatments. Here, we evaluated the effect of targeting the brain tumor microenvironment via the myeloid colony-stimulating factor-1 receptor (CSF-1R) pathway using the small molecule inhibitor BLZ945. Studies were conducted in two TNBC hematogenous brain-tropic models, 4T1-BR5 and 231-BR, with endpoints of prevention of brain metastasis formation and treatment of established brain metastasis. BLZ945 reduced the formation of brain metastases in both models by 57–65% (all *p* < 0.01) in the prevention setting. In the treatment setting, more analogous to the clinical situation, BLZ945 reduced the number and size of metastases in both models by 44–65% and 61–72%, respectively (all *p* < 0.05). Treatment with BLZ945 significantly reduced the number of myeloid cells in both the uninvolved brain and metastatic regions, by 15–54% across models as early as three days post-treatment. Efficacy was achieved without the need for complete suppression of brain myeloid cells, suggesting that potential adverse effects of full myeloid suppression can be minimized. Additionally, BLZ945 reduced cancer cell proliferation and astrocyte activation in the tumor microenvironment *in vivo*. *In vitro* studies showed that BLZ945 inhibited the secretion of inflammatory cytokines that stimulated cancer cell invasion; BLZ945 also indirectly reduced cancer cell proliferation through astrocyte interaction. Our findings suggest that microglial CSF-1R controls a series of myeloid regulatory pathways, both alone and in concert with other brain microenvironmental cells. The data preclinically credential CSF-1R inhibition as a potential therapeutic strategy for TNBC brain metastases.

**Supplementary Information:**

The online version contains supplementary material available at 10.1007/s10585-025-10366-x.

## Introduction

Brain metastases occur frequently in breast cancer patients with metastatic HER2 overexpressing (HER2^+^) or triple-negative disease. Standard treatments include stereotactic radiosurgery to individual lesions, whole brain radiotherapy for widespread disease, surgery, chemotherapy and steroids. HER2^+^ brain metastases often occur as an early relapse in metastatic treatment when systemic lesions are often stable or responding [1]. HER2 kinase inhibitors or antibody-based therapies have demonstrated efficacy for brain metastases in terms of progression-free survival (PFS) and overall survival (OS) [2–4]. Metastatic TNBC cells harbor none of the accepted breast cancer biomarkers (HER2 amplification/overexpression, estrogen receptor, progesterone receptor). Metastatic TNBC patients develop brain metastases at a high frequency, often in the context of uncontrolled systemic disease. Median survival for TNBC patients after brain metastases is approximately 5 months [5].

Therapies targeting either tumor cell molecular pathways or the immune checkpoint system have been the mainstay of clinical-translational research for TNBC and accompanying brain metastases. Recent trials have demonstrated a progression-free survival advantage for the Trop-2-directed antibody–drug conjugate (ADC) sacituzumab govitecan in metastatic TNBC [6]; a post-hoc analysis of 61 patients enrolled with stable brain metastases showed a PFS of only 2.6 versus 1.8 months on the chemotherapy arm, with no difference in overall survival [7]. The combination of pembrolizumab and investigator’s choice of chemotherapy significantly prolonged overall survival compared to chemotherapy alone in patients with advanced TNBC [8, 9]. Trastuzumab deruxtecan (T-DXd) is a highly effective targeted therapy for HER2-positive breast cancer. It has been approved in the United States for the treatment of adult patients with unresectable or metastatic hormone receptor (HR)-negative, HER2-low breast cancer, and more recently, for those with unresectable or metastatic HR-positive, HER2-low or HER2-ultralow breast cancer, some of whom would be considered as triple-negative [10, 11]. It has shown promising preliminary activity, both intra- and extracranially, in pretreated HER2-low advanced breast cancer patients with active brain metastases [12].

Because of the complex and heterogeneous mutational and gene expression patterns in advanced cancers such as brain metastases, a distinct therapeutic strategy is to target the tumor microenvironment [13]. Multiple cell types are present in brain metastases: In a normal brain, neurons are ensheathed by myelin produced by oligodendrocytes. The permeability of the capillary network is limited by the blood–brain barrier, which consists of endothelial cells, pericytes, astrocytes, and basement membranes. In addition to their role in maintaining the blood–brain barrier, astrocytes support synapse formation and function, and regulate neurotransmitter clearance. Microglia are long-lived, phagocytic myeloid cells that provide innate immune surveillance, and prune neural connections. As metastases develop, the brain microenvironment is locally altered, including neuronal death, development of a neuroinflammatory response consisting of activated microglia and GFAP^+^ astrocytes, altered permeability of the now blood-tumor barrier and limited influx of systemic immune cells including bone marrow-derived macrophages.

Several labs have hypothesized that microglia/infiltrating macrophages in the microenvironment could serve as regulatory points for brain metastases [14–16]. This hypothesis is greatly complicated by the fact that microglia are functionally heterogeneous, in both the normal and disease states, and with age. Microglia/macrophages were originally classified as pro-inflammatory type I and anti-inflammatory type II, but recent sequencing analysis of normal and brain metastasis-associated myeloid cells has identified multiple operative functional pathways including cytokine production, interferon responses, leukocyte migration, and interactions with extracellular matrix [17, 18]. The use of any single microglial/macrophage modulator is therefore expected to depend on the relative presence and activity of these multiple functions.

Brain microglia and macrophages are increasingly recognized as active participants in the brain metastasis. Depletion experiments provided initial evidence for a role of microglia/macrophages in brain metastasis. Depletion of brain myeloid cells using Cx3cr1^CreERT/+^:ROSA26^iDTR/+^ mice significantly impaired the formation of E0771 triple negative breast cancer brain metastasis formation if cancer cells were injected after gene knockout, but not when colonization and gene knockout occurred simultaneously [18]. This effect has been attributed to microglia as similar experiments in Ccr2^−/−^ and Ccr2^+/–^ mice with impaired monocyte infiltration did not affect brain metastasis [18]. Mannosylated clodronate liposomes were also tested as a general microglia/macrophage depletion scheme and reduced the area of intracranially injected TNBC 4T1 breast cancer cells [19]. In contrast, a genetic knockout model that depleted microglia impaired NK and T-cell responses and resulted in decreased rejection of E0771 TNBC intracranial implants and increased tumor growth [20].

A potentially druggable point of microglial/macrophage regulation is the colony stimulating factor-1 (CSF-1) receptor (CSF-1R) which binds both CSF-1 and IL-34. This tyrosine kinase receptor regulates the viability, growth and differentiation of microglia [21]. IL-34 is also abundant in the brain and regulates microglia through CSF-1R [22] as well as other receptors. Macrophages also proliferate and differentiate in response to CSF-1 [23]. Limited data are available on the antitumor activity of CSF-1R inhibition in TNBC brain metastases. Klemm et al*.* used the brain-permeable sotuletinib (BLZ945) CSF-1R inhibitor in pretreatment models of hematogenous MDA-MB-231-BrM triple-negative and 99LN-BrM luminal B (estrogen receptor positive, HER2 overexpressed) brain metastasis models, which altered microglial localization away from tumor cells and extended survival [24]. In hematogenous intervention models, in which BLZ945 was administered after brain metastasis formation, reductions in tumor size by BLZ945 were more prominent in the triple negative model than the luminal B model, and some tumor regrowth was observed after longer treatment [24]. The Sibson lab reported that the M279 monoclonal antibody against CSF-1R inhibited the growth of intracranially inoculated TNBC tumor cells, but the effect was predominantly due to infiltrating macrophages [25]. We previously studied the effect of mouse age on brain metastasis formation and found that older mice developed 2-4 fold fewer hematogenous brain metastases than younger mice across three models of TNBC [26]. CNS myeloid cells contributed both to the development of TNBC brain metastases and to the observed age-related differences in metastasis formation. A brain-permeable CSF-1R inhibitor, pexidartinib (PLX3397) was administered to mice before injection of 4T1-BR5 triple negative breast cancer cells, and significantly reduced brain metastases in young mice [26]. CSF-1R has also been characterized in glioma progression [27].

Although these data suggest potential for CSF-1R inhibition, several important variables remain unresolved, including: (1) intracranial injection of tumor cells versus hematogenous metastasis assays; (2) differential responsiveness of breast cancer subtypes to CSF-1R inhibition; (3) the potency and specificity of CSF-1R inhibitors; (4) timing and frequency of drug administration; (5) endpoints of experiments (prevention, treatment of established lesions). Here, we have used BLZ945 as a CSF-1R inhibitor, based on its specificity and potency, in two hematogenous model systems of TNBC brain metastasis. Using doses and schedules that mimic the preventive and established metastatic settings, we report the efficacy of BLZ945 on both metastasis number and size. Interestingly, efficacy was achieved without complete or near-complete suppression of CNS myeloid cells. Supporting *in vitro *data suggest a complex mechanism of action based on microglial/macrophage cytokine secretion to influence tumor cell invasion and astrocytic activation.

## Materials and methods

### Animal experiments and CSF-1R inhibitor

All animal procedures were approved by the NCI-Frederick ACUC. NCI-Frederick is accredited by AAALAC International and follows the Public Health Service Policy for the Care and Use of Laboratory Animals. Animal care was provided in accordance with the procedures outlined in the “Guide for Care and Use of Laboratory Animals (National Research Council; 1996; National Academy Press; Washington, D.C.). For breast cancer brain metastasis models, cancer cells suspended in 100 µL sterile PBS were injected into the left ventricle of mice under isoflurane anesthesia as previously described [26]. Specifically, 5 × 10^4^ 4T1-BR5 cells and 1.75 × 10^5^ 231-BR cells were injected into female BALB/c (4 months old) and athymic nude mice (4 months old) respectively. BLZ945 was obtained under a Material Transfer Agreement from Novartis. Immediately prior to the experiment, BLZ945 was dissolved in 20% Captisol^®^ at 20 mg/ml. BLZ945 was administered daily by oral gavage at a dose of 200 mg/kg according to previous studies [24, 28].

### Cell assays

Cell culture conditions and generation of bone marrow-derived macrophages (BMDM) are described in the Supplementary Methods section. Cell viability assay was performed with AlamarBlue according to the manufacturer’s protocol (Thermo Fisher Scientific). All cells, except BMDM, were seeded at 1 × 10^3^ cells per well in black Corning 96-well plates with clear bottom and cultured in cell culture medium supplemented with increasing doses of BLZ945 or DMSO for 72 h. BMDM was seeded at 2.5 × 10^3^ cells per well and treated with BLZ945. To measure cell proliferation, 1 × 10^3^ cancer cells were plated in black, clear-bottom Corning 96-well plates. The cells were cultured in different conditioned cell culture media for 72 h, followed by an AlamarBlue assay. The fluorescence signal was measured under a fluorimeter (excitation at 550 nm and emission at 590 nm) using a SpectraMax ID3 microplate reader (Molecular Devices). The fluorescence signal was normalized to the vehicle control treated cells.

### Western blot

Cells were lysed in cell lysis buffer (20 mM Tris-HCl, pH 7.5, 150 mM NaCl, 1% sodium deoxycholate, 1 mM EDTA, 1 mM EGTA, 2.5 mM sodium pyrophosphate, 1 mM sodium orthovanadate, 1% NP-40) with protease and phosphatase inhibitors (Thermo Fisher Scientific). Overall, 20 μg protein was resolved on 4–12% Bis-Tris gels and transferred to polyvinylidene difluoride (PVDF) membranes. Additional details are provided in the Supplementary Methods section.

### Mouse cytokine antibody array

The mouse cytokine antibody array (#ARY006, R&D Systems) was used according to the manufacturer's instructions and analyzed using the ImageJ software. The identity and the respective coordinates of all the antibodies on the arrays are available on the manufacturer’s website.

### Brain metastasis experiments and tissue collection

For brain metastasis models, mice were anesthetized with isoflurane and transcardially perfused with cold Krebs–Ringer solution at the end of the experiment (13 days for 4T1-BR5 and 27 days for 231-BR after intracardiac injection of cancer cells). The brains were then collected and bisected. The right hemispheres were immediately snap frozen in OCT (i.e., fresh frozen) and the left hemispheres were fixed in 4% paraformaldehyde (PFA) for 4 h at 4 °C. The fixed brain tissues were then transferred to 20% sucrose for 24 h at 4 °C, and embedded in OCT (i.e., fixed frozen). Brain tissues were stored at − 80 °C until processed.

To quantify the brain metastatic tumor burden, OCT-embedded mouse left brain hemispheres were histologically sectioned into five spatially separated planes (approximately 500 µm between 2 adjacent sagittal planes). At each of the five depths, five 8 µm thick sections were cut by a cryostat. One slide was used for H&E staining to quantify the tumor burden, and the others were used for immunofluorescence staining. Representative H&E-stained slides were scanned using a Morphle Optimus 6 × slide scanner, and images were further analyzed using Morphle software. Brain metastatic tumor burden was quantified as number per section, and metastatic area was quantified in µm^2^ by hand-drawing lesion boundaries through one brain hemisphere following previously established protocols [26].

### Immunofluorescence staining and graphic representation

Immunofluorescence staining was performed on 8-μm thick PFA-fixed, OCT-embedded tissue sections. Additional details are provided in the Supplementary Methods section.

### Statistical analysis

Analysis of step sections for number and size of metastases is reported on the basis of individual animals. Immunofluorescence imaging data are presented as the intensity of 4–5 representative images from each group. Statistical analysis was performed using GraphPad software. The Mann–Whitney U test was used to compare unpaired data. *p* values < 0.05 were considered statistically significant.

## Results

### CSF-1R inhibition reduces only microglial and macrophage cell proliferation *in vitro*

**Fig. 1:**
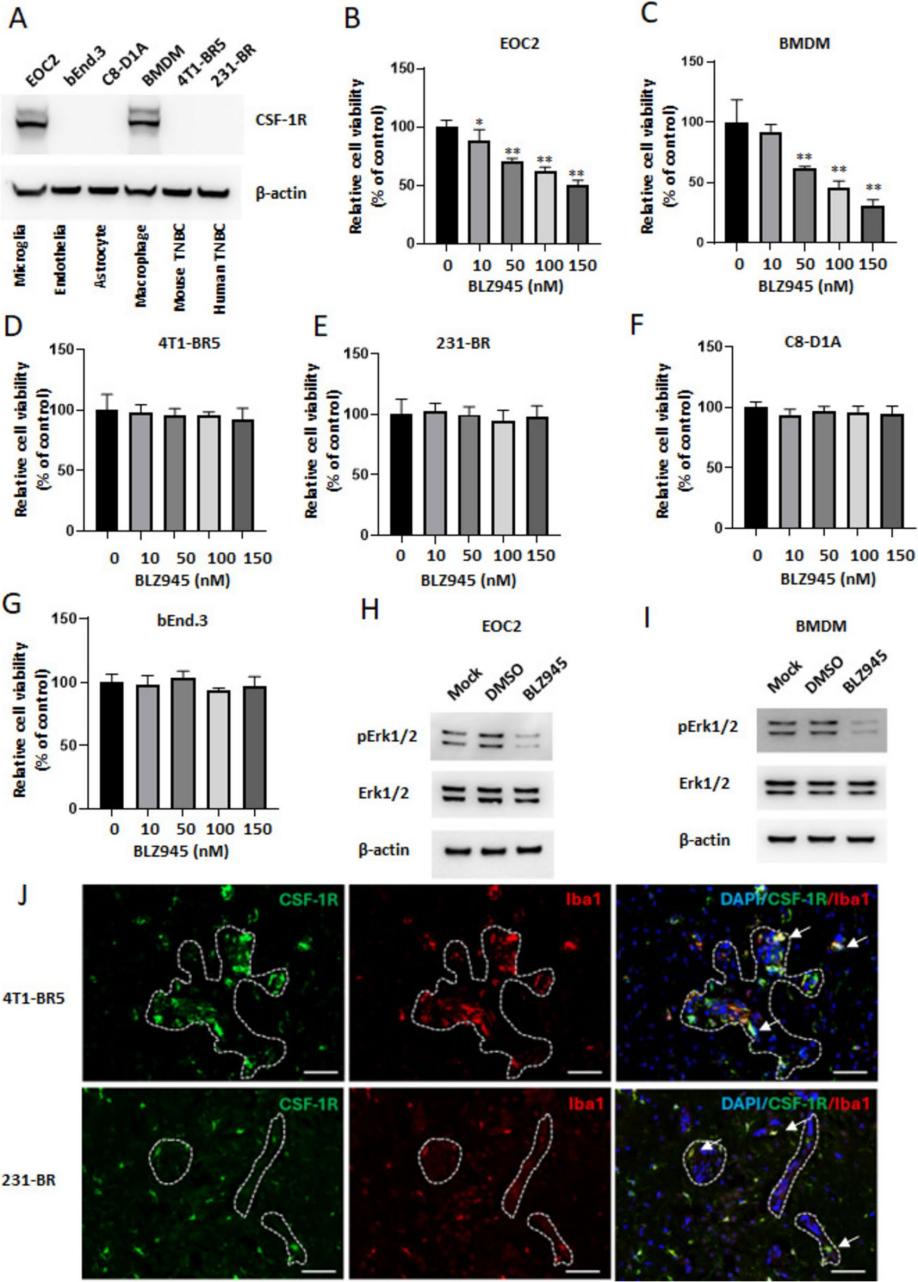
CSF-1R expression is limited to brain microglia/macrophages. **A** Western blot analysis of CSF-1R in EOC2 (microglia), bEnd.3 (endothelia), C8-D1A (astrocyte), BMDM (primary macrophage), 4T1-BR5 (mouse TNBC) and 231-BR (human TNBC). β-actin is used as a loading control. **B**–**G** EOC2, BMDM, 4T1-BR5, 231-BR, C8-D1A or bEnd.3 cells were exposed to dimethyl sulfoxide (DMSO, vehicle control) or BLZ945 (10, 50, 100 and150 nM) for 72 h followed by alamarBlue cell viability assay. Values represent the mean fold change ± SEM relative to vehicle control (n = 6). **H**–**I** Western blot analysis of pErk1/2, Erk and β-actin protein in EOC2 and BMDM cells treated with BLZ945 (EOC2, 150 nM; BMDM, 100 nM) or DMSO for 48 h. *, *p* < 0.05 vs DMSO control; **, *p* < 0.01 vs DMSO control by Mann–Whitney test. **J** CSF-1R (green) co-staining with Iba1 (red) in frozen, formaldehyde-fixed and OCT-embedded metastases and their microenvironments of 4T1-BR5 (upper panel) and 231-BR (lower panel) mouse models of breast cancer brain metastasis. Arrows indicate examples of co-stained cells, and white dotted lines indicate brain metastases. All nuclei are stained with DAPI (blue). Scale bar = 50 μm for all panels

Since BLZ945 is a highly selective, brain-penetrating, potent kinase inhibitor of CSF-1R, we first measured the level of CSF-1R protein by Western blot in mouse cell lines from brain-derived microglia (EOC2), endothelia (bEnd.3), astrocytes (C8-D1A), bone marrow-derived macrophages (BMDM), and two brain-tropic TNBC lines (mouse 4T1-BR5 and human 231-BR). CSF-1R was expressed exclusively in EOC2 microglial cells and BMDM (Fig. 1A). Cell viability assays were then performed using BLZ945 at concentrations ranging from 10–150 nM for 3 days. BLZ945 showed an anti-proliferative effect on both EOC2 and BMDM in a dose-dependent manner (Fig. 1B and C). The half-maximal inhibitory concentration (IC50) of BLZ945 for inhibiting CSF-1R-dependent cell survival was 142 nM for EOC2 and 98 nM for BMDM, consistent with previous studies [28]. In contrast. BLZ945 had no obvious inhibitory effects on astrocytes, endothelia and 4T1-BR5 or 231-BR cancer cells *in vitro* (Fig. 1D–G) in agreement with their lack of CSF-1R expression. Inhibition of microglia/macrophage proliferation was accompanied by decreased Erk phosphorylation (Fig. 1H and I).

In metastatic mouse brain sections, CSF-1R distribution coincided with Iba1 staining for microglia and infiltrating macrophages (Fig. 1J), but not GFAP for astrocytes (Supplementary Fig. S1), in both the 4T1-BR5 and 231-BR models, confirming the cell line data. CSF-1R^+^ microglia/macrophages prominently surrounded and infiltrated metastatic lesions as part of the neuroinflammatory response, in addition to residing in the uninvolved brain (Fig. 1J). These data confirm that in the models used, microglia and infiltrating macrophages were the only discernable direct targets of BLZ945.

### CSF-1R inhibition suppresses brain metastasis in the prevention and treatment settings in the 4T1-BR5 TNBC model

**Fig. 2:**
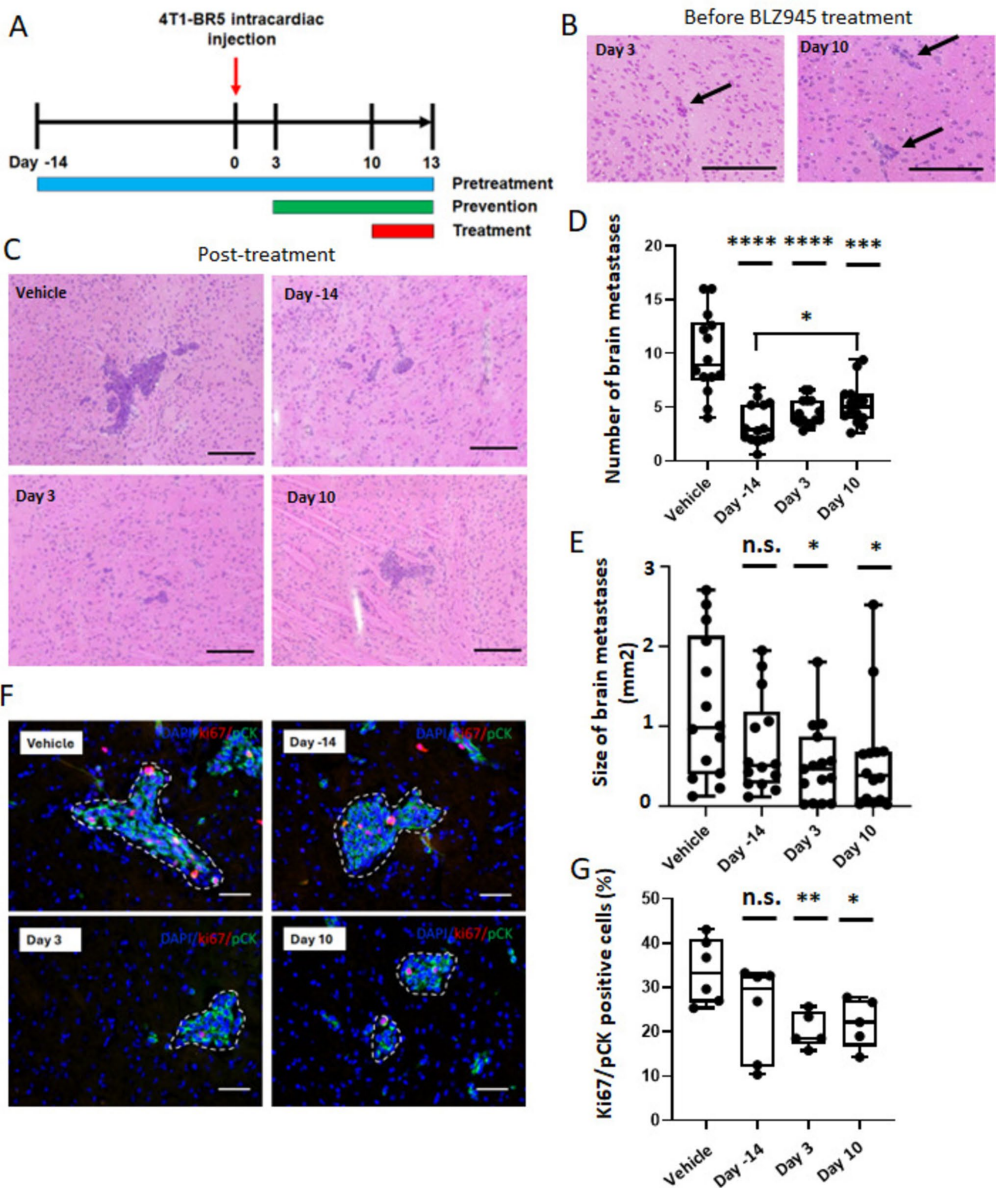
CSF-1R suppression in the prevention and treatment settings of 4T1-BR5 TNBC brain metastasis model. **A** Schematic representation of the pretreatment, prevention and treatment studies. **B** Representative hematoxylin and eosin (H&E) staining images of 4T1-BR5 cancer cells in the brain after intracardiac injection at the start of the prevention (day 3) and treatment (day 10) arms. Arrows indicate metastatic cancer cells. **C** Representative H&E staining images of 4T1-BR5 brain metastases on day 13 after vehicle or BLZ945 treatment at the endpoint. Scale bar = 200 μm for panel B & C. **D** Number of brain metastases per brain section. **E** Size of brain metastases per animal. *n* = 14–15. **F** Representative immunofluorescence staining images of Ki67 (red) and pCK (green) in brain metastases (white dotted line) from vehicle and BLZ945 treated mice. Scale bar = 50 μm. **G** Quantification of Ki67 and pCK-positive cells in metastases from 5 to 6 biological replicates. Each dot represents one mouse, and the line designates the group median. Statistical significance versus vehicle control was calculated using the Mann–Whitney test (*, *p* < 0.05; **, *p* < 0.01; ***, *p* < 0.001; ****, *p* < 0.0001 vs vehicle control; n.s., not significant)

We evaluated the efficacy of BLZ945 in a well-characterized TNBC brain metastasis model. The 4T1-BR5 line was derived from the murine triple-negative breast cancer 4T1 cells, which were selected via multiple rounds of intracardiac injection and harvesting of metastatic tumor cells in the brain *in vivo* for increased ability to develop brain metastases [29]. An advantage of this model is its immunocompetence. Previous studies have shown that daily oral gavage of 200 mg/kg of BLZ945 significantly reduced tumor growth [28]. As illustrated in Fig. 2A, BALB/c mice were randomized into three BLZ945 (200 mg/kg/day by oral gavage) and one vehicle control (20% captisol) arms. In the first BLZ945 group, mice were treated with drug 14 days prior to intracardiac injection of 4T1-BR5 cells, and dosing continued throughout the experiment (pretreatment group). This schema was based on prior reports using a pretreatment schema but has no translational relevance [24, 26]. In the prevention and treatment groups, mice were treated orally with BLZ945 daily starting on day 3 post-injection and day 10 post-injection, respectively, and continued treatment until day 13 endpoint. Body weight and general health of BALB/c mice were monitored during treatment and no serious toxicity was observed in either the control or treatment groups (Supplementary Fig. S2). Representative photographs of brain sections from mice sacrificed on days 3 and 10 before treatment and on day 13 after vehicle or BLZ945 treatment are shown in Fig. 2B and C, respectively. The micrometastases on days 3 and 10, prior to treatment, were also validated using pan-cytokeratin (pCK) staining, a marker for cancer cells, and CD31, an endothelial cell marker (Supplementary Fig. S3A).

A 67% decrease in the median number of metastases was observed in the pretreatment arm (*p* < 0.0001). Notably, a similar 57% reduction in metastases was observed in the prevention-of-metastasis-formation arm (*p* < 0.0001) and a 44% reduction was observed in the treatment-of-established-metastases arm (*p* < 0.001) (Fig. 2D). The reduced number of brain metastases was also accompanied by changes in metastases size (Fig. 2E). Pretreatment with BLZ945 resulted in a 48% reduction in median metastases size (*p* = 0.18). The prevention arm resulted in a stronger 52% reduction in median size (*p* < 0.05), while the treatment arms, consisting of only three doses of BLZ945, resulted in comparable results, a 61% reduction in median size (*p* < 0.05). Among the three treatment settings, the pretreatment group showed a significant reduction (42%) in metastases number compared to the treatment setting (Fig. 2D). There was no significant difference in the size of metastases among the three treatment groups (Fig. 2E).

To exclude the possibility of counting infiltrated myeloid cells, we performed Ki67 staining along with pCK staining. The reduction in brain metastasis number and size by BLZ945 treatment was accompanied by reduced tumor cell proliferation (Ki67/pCK double positive, Fig. 2F and G). The median Ki67/pCK staining positivity was 89% (not significant, NS), 56% (*p* < 0.01) and 67% (*p* < 0.05) of control levels in the pretreatment, prevention, and treatment arms respectively. The reduction in proliferation was not due to cell death as cleaved caspase 3 (CC3) staining showed no significant trends (Supplementary Fig. S4). In addition, BLZ945 treatment did not inhibit angiogenesis, in terms of CD31^+^ microvascular density (Supplementary Fig. S5).

**Fig. 3:**
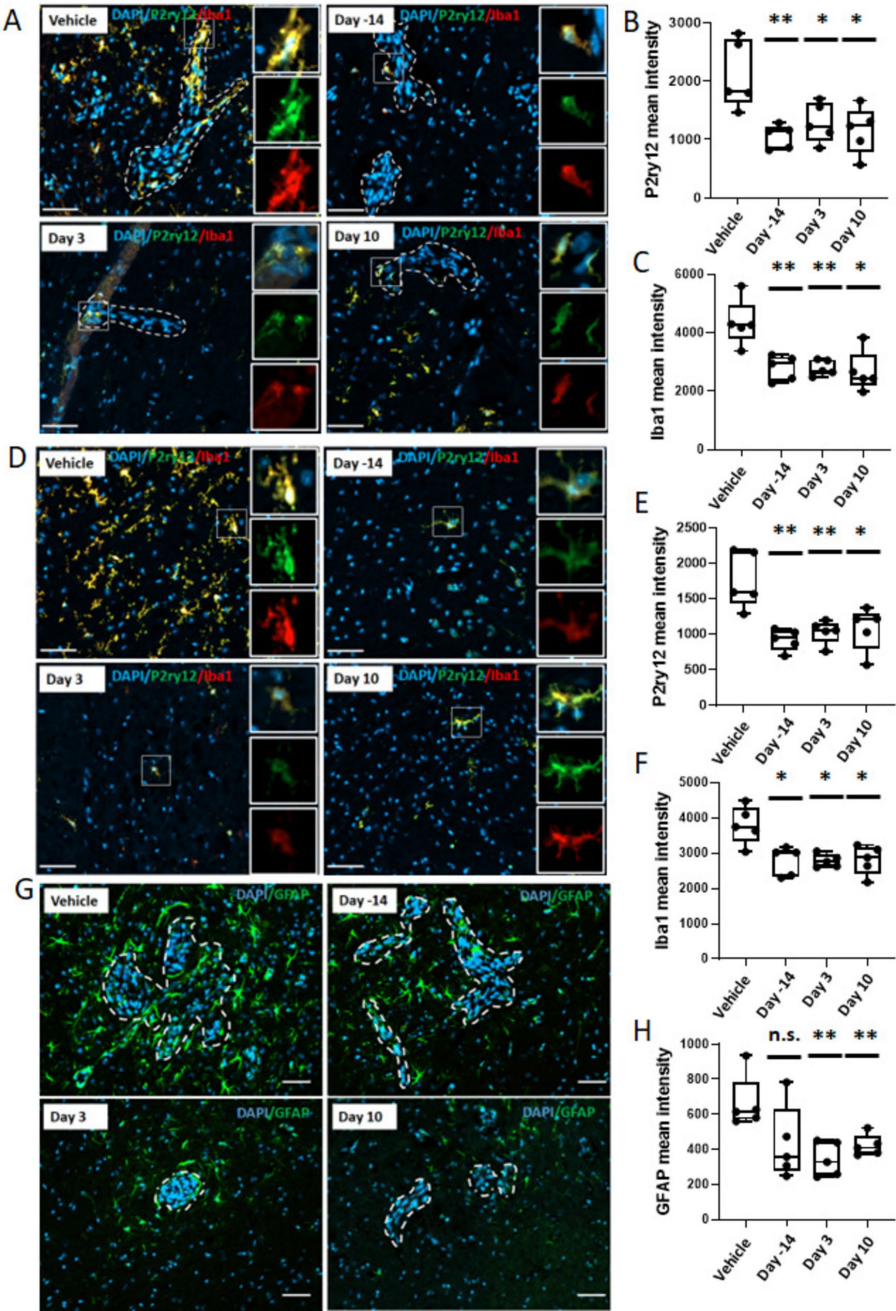
Effect of CSF-1R suppression on microglia/infiltrating monocytes and astrocyte activation in 4T1-BR5 model. Representative immunofluorescence images and quantification of P2ry12 (green) and Iba1 (red) staining in brain metastases (**A**–**C**) and uninvolved brain tissues (**D**–**F**) in control and BLZ945-treated mice at the endpoint. **G** and **H** Representative immunofluorescence staining images and quantification of GFAP (green) in the tumor microenvironment in control and BLZ945-treated mice (*n* = 5). All nuclei are stained with DAPI (blue). Each dot represents one mouse, and the line designates the group median. Scale bar = 50 μm. *, *p* < 0.05; **, *p* < 0.01 vs vehicle control; n.s., not significant by the Mann–Whitney test

To investigate whether BLZ945 hit its intended target, immunofluorescent staining was performed on microglial and infiltrating macrophage populations both in the metastatic region (Fig. 3A–C) and uninvolved brain (Fig. 3D–F). P2ry12, a P2 purinergic receptor, is considered a specific marker for rodent microglial cells under normal and pathological conditions, while Iba1 is a well-established dual marker for both microglia and macrophages [26]. In the brain metastases all three BLZ945 arms similarly reduced the P2ry12^+^ microglial population by 32–37% (all *p* < 0.05). Similar results were obtained for combined myeloid populations by Iba1 staining with a 31% reduction in the pretreatment arm (*p* < 0.01), 37% reduction in the prevention arm (*p* < 0.01) and 43% reduction in the treatment arm (*p* < 0.05). In the uninvolved brain myeloid populations were also reduced, by 41%, 34% and 24% in the pretreatment, prevention and treatment arms, respectively, using P2ry12 (all *p* < 0.05) and 20%, 26% and 23% respectively using Iba1 (all *p* < 0.05). To further confirm, CSF-1R^+^ cells in the brains of mice from the four arms were also evaluated by immunohistochemistry. Reductions in the number of stained cells were observed in all treatment arms in the metastatic microenvironment and uninvolved brain (Supplementary Fig. S6).

Myeloid-derived suppressor cells or neutrophils may also play a role in the development of brain metastasis [30]. To assess whether BLZ945 targeted these cells, we performed myeloperoxidase (MPO) staining. While MPO^+^ cells were readily detected in a 4T1-derived xenograft, they were scarcely detected in the brain metastases, regardless of BLZ945 treatment (see Supplementary Fig. S7A-B). This suggests that myeloid-derived suppressor cells or neutrophils may not play a major role in the metastasis suppression mediated by BLZ945.

Although BLZ945 had no antiproliferative effect on an astrocytic cell line *in vitro* (Fig. 1F), in staining for the extent of the neuroinflammatory response, we noted a prominent reduction in astrocyte activation in the metastatic microenvironment *in vivo*. Figures 3G and H show staining for activated astrocytes (GFAP^+^) in the control and treatment groups and quantification of staining intensity. BLZ945 treatment reduced GFAP by 42% in the pretreatment group (not significant), 47% in the prevention group (*p* < 0.01) and 34% in the treatment group (*p* < 0.01). Astrocytes in the prevention and treatment groups were also smaller with fewer extensions. The data suggest the intriguing possibility that inhibition of astrocytic activation may be downstream of CSF-1R^+^ myeloid cells in this model.

We previously reported that young mice developed 2-4 fold greater brain metastases than aged mice in triple-negative and luminal B model systems, and that a CSF-1R inhibitor PLX3397 showed better metastasis preventive activity in the younger cohort [26]. The experiment was repeated using 4T1-BR5 brain-tropic tumor cells and syngeneic BALB/c mice at 7 and 18 months of age. Young mice in the vehicle control arm again developed greater numbers of brain metastases than aged mice (Supplementary Fig. S8). BLZ945 reduced the median number of brain metastases in all arms of the experiment although it only obtained statistical significance in the young cohort in a prevention setting (Supplementary Fig. S9). The interpretation of the data is complicated by the poor survival of old mice.

In summary, in the 4T1-BR model system, BLZ945 significantly inhibited both brain metastasis outgrowth (prevention setting) and growth of established lesions (treatment setting). The latter consisted of only three daily doses, is more comparable to a traditional clinical trial design. Its inhibitory effects were accompanied by a partial reduction in microglia/macrophages, but neither a prevention nor a treatment effect required the full elimination of microglia/infiltrating macrophages.

### CSF-1R inhibition suppresses brain metastasis in the prevention and treatment settings in the 231-BR TNBC model

**Fig. 4:**
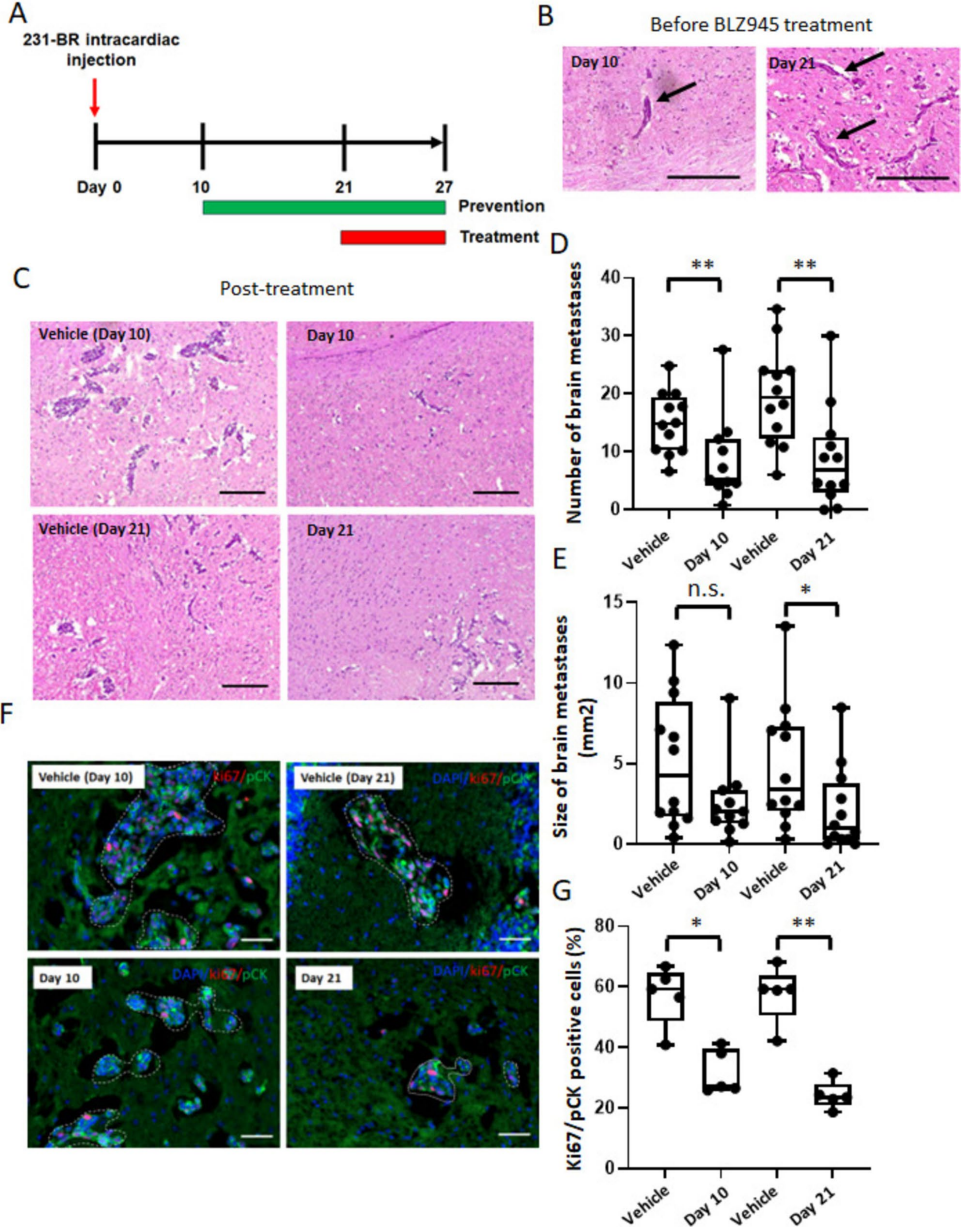
CSF-1R suppression in the prevention and treatment settings in 231-BR TNBC brain metastasis model. **A** Schematic representation of the prevention and treatment studies. **B** Representative H&E staining images of 231-BR cancer cells in the brain after intracardiac injection at the start of the prevention (day 10) and treatment (day 21) arms. Arrows indicate metastatic cancer cells. **C** Representative H&E staining images of 231-BR brain metastases on day 27 after vehicle or BLZ945 treatment at the endpoint. Scale bar = 200 μm for panels B&C. **D** and **E** Number of brain metastases per brain section and size of brain metastases per animal. Each dot represents one mouse, and the line designates the group median. *n* = 11–12. **F** and **G** Representative immunofluorescence staining images and quantification of Ki67 and pCK-positive cells in brain metastases (white dotted line) from vehicle and BLZ945 treated mice (*n* = 5). Scale bar = 50 μm. *, *p* < 0.05; **, *p* < 0.01 vs vehicle control; n.s., not significant by the Mann–Whitney test

To confirm the relevance of CSF-1R inhibition to TNBC, we tested the ability of BLZ945 to prevent and treat brain metastasis in a second hematogenous brain metastasis model system. A brain-tropic subline of the human TNBC 231-BR cell line was used to generate experimental brain metastases in immunocompromised nude mice [31]. This model requires 27 days, with a prevention arm beginning dosing on day 10 post-injection and a treatment arm beginning on day 21 post-injection, the latter delivering only six daily doses to established lesions; each arm had a vehicle arm comparator with the same schedule (Fig. 4A). Representative lesions from untreated mice on days 10 and 21 post-injection are shown in Fig. 4B and Supplementary Fig. S3B. Representative lesions from each experimental arm are shown on Fig. 4C and lesion number and size graphed on Fig. 4D and E. A pretreatment arm was not performed as it is translationally irrelevant. Nude mouse mean body weight and general health were followed, and no severe toxicity was observed in either control or treatment groups (Supplementary Fig. S10).

Efficacy in both the treatment and prevention arms was confirmed in the 231-BR model. A notable 65% reduction in median metastasis number was observed in the prevention arm (*p* < 0.01) and an equivalent 65% reduction was observed in the treatment arm (*p* < 0.01). The prevention arm resulted in a 52% reduction in median metastasis size (NS), while the treatment arms resulted in a 72% reduction in median size (*p* < 0.05). There was no significant difference in the number or size of metastases between the two treatment groups.

Both arms resulted in decreased Ki67/pCK positive tumor cells, 54% in the prevention arm (*p* < 0.05) and 60% in the treatment arm (*p* < 0.01) (Fig. 4F and G). Apoptosis, as measured by the expression of CC3, was not significantly affected in either arm (Supplementary Fig. S11). Consistent with the 4T1-BR5 model, BLZ945 treatment did not affect angiogenesis in the 231-BR model (Supplementary Fig. S12).

**Fig. 5:**
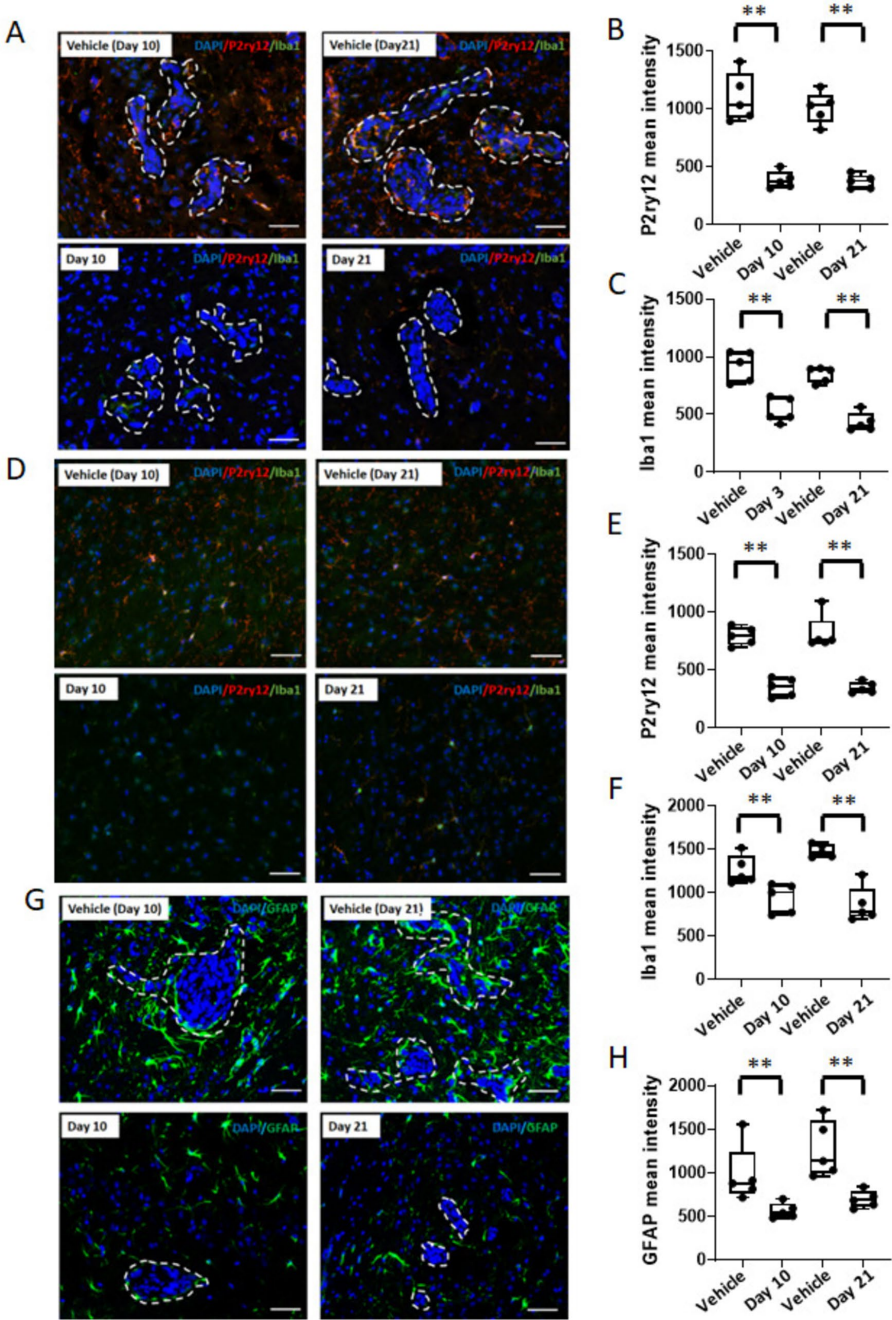
Effect of CSF-1R suppression on microglia/infiltrating monocytes and astrocyte activation in 231-BR model. Representative immunofluorescence images and quantification of P2ry12 (red) and Iba1 (green) staining in brain metastases (**A**–**C**) and uninvolved brain tissues (**D**–**F**) in control and BLZ945-treated mice (*n* = 5) at the endpoint. **G** and **H**, Representative immunofluorescence staining images and quantification of GFAP (green) in the tumor microenvironment in control and BLZ945-treated mice (*n* = 5). All nuclei are stained with DAPI (blue). Each dot represents one mouse, and the line designates the group median. Scale bar = 50 μm. **, *p* < 0.01 vs vehicle control; n.s., not significant by the Mann–Whitney test

Immunofluorescent staining of the myeloid components of the metastatic microenvironment (Fig. 5A–C) and uninvolved brain (Fig. 5D–F) confirmed an inhibitory effect of BLZ945. In the metastatic region experimental arms similarly reduced the P2ry12^+^ microglial population by approximately 60% (all *p* < 0.01). Similar results were obtained for all myeloid populations using Iba1 staining with a 49% reduction in the prevention arm (*p* < 0.01) and 54% reduction in the treatment arm (*p* < 0.01). In the uninvolved brain myeloid populations were also reduced by 54% and 56% in the prevention and treatment arms, respectively, using P2ry12 (all *p* < 0.01) and 15%, and 47% respectively using Iba1 (all *p* < 0.01). The effect of BLZ945 on astrocyte activation was also confirmed in the metastatic microenvironment in the 231-BR model system (Fig. 5G and H). BLZ945 treatment reduced GFAP by 39% in the prevention group (*p* < 0.01) and 40% in the treatment group (*p* < 0.01).

The data indicate that CSF-1R inhibition has both preventive and treatment efficacy in TNBC brain metastases. Both mouse 4T1-BR5 and human 231-BR models showed efficacy with only partial ablation of microglia. Both models showed that the drug hit its intended targets and confirm that the phenotype is not limited to myeloid cells but also includes reduced activation of astrocytes.

### CSF-1R inhibition alters microglial cytokine secretion

**Fig. 6:**
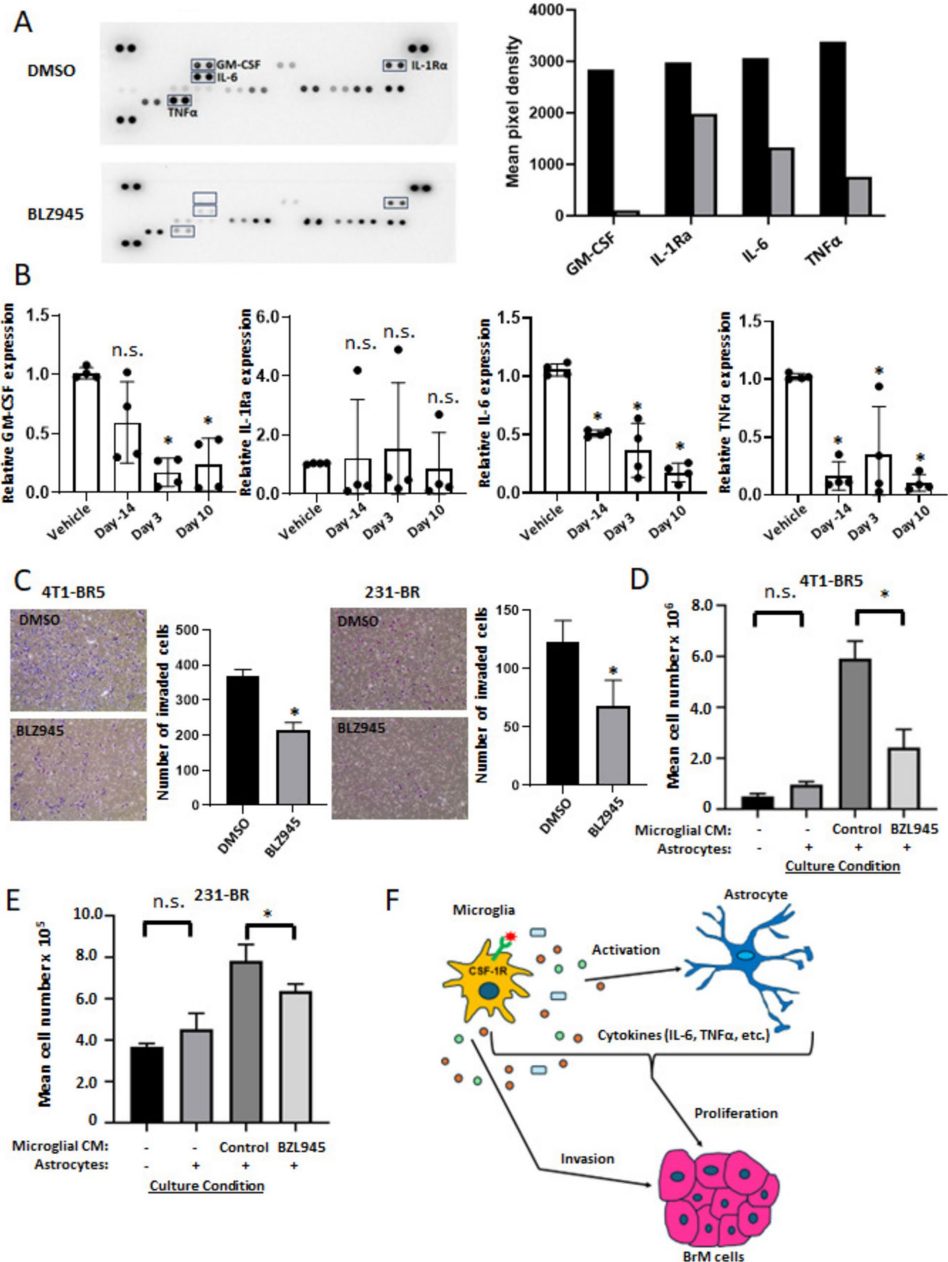
CSF-1R suppression alters microglial cytokine secretion patterns with effects on astrocytes and tumor cells. **A** Mouse cytokine antibody array analysis of EOC2-derived conditioned medium treated with 100 nM BLZ945 or DMSO for 72 h. The images of cytokines with marked GM-CSF, IL-1Rα, IL-6 and TNFα are shown in the left panel. The pixel density of the above cytokines relative to the internal control is shown by the graphs in the right panel. **B** RT-PCR analysis of *GM-CSF*, *IL-1Rα*, *IL-6*, and *TNFα* was performed on fixed, frozen specimens from vehicle control and BLZ945 treatment settings of the 4T1-BR5 TNBC brain metastasis model mice on Days -14, 3, and 10. Values represent the fold change ± SD relative to vehicle. Each dot represents one mouse, and the line designates the group median. **C** 4T1-BR5 and 231-BR cells were allowed to invade through Matrigel for 12 h in the presence of control or BLZ945 treated EOC2-derived conditioned medium. Invaded cells were visualized by Hema 3 staining and counted using ImageJ software. **D **and **E** EOC2 conditioned medium promotes the growth of 4T1-BR5 and 231-BR cells under co-culture conditions. 4T1-BR5 and 231-BR cells were labelled with mCherry and EGFP, respectively. Brain trophic cancer cell numbers are quantified and calculated by flow cytometry based on the ratio between cancer cells and quantitative beads. The effect of astrocytes alone on cancer cell growth was measured in low serum medium under co-culture conditions. *n* ≥ 3 biologically independent experiments. *, *p* < 0.05 vs DMSO control; n.s., not significant by the Mann–Whitney test. **F** Schematic representation of microglial action in breast cancer brain metastasis. CSF-1R^+^ microglia/macrophages regulate the secretion of cytokines that influence the behavior of astrocytes and brain metastatic (BrM) cells within the microenvironment

Our experiments confirmed the expected decrease in microglial/macrophage abundance with BLZ945 treatment, but also identified an unexpected decrease in astrocyte activation, given the lack of CSF-1R receptors on these cells. We hypothesized that this may result from BLZ-945 alteration of microglia/infiltrating macrophage secretion patterns that ultimately affect astrocytes. To test this hypothesis, an antibody-based cytokine array quantified levels of cytokines in the conditioned culture medium of EOC2 microglia treated with 100 nM BLZ945 or DMSO for 3 days (Fig. 6A). Of the more than 40 mouse cytokines and chemokines evaluated, reductions in GM-CSF, interleukin-1 receptor antagonist (IL-1Ra), IL-6 and TNFα were observed in response to BLZ945 treatment (Fig. 6A). To confirm the cytokine array results, transcriptional expression levels of *GM-CSF, IL-1Ra, IL-6*, and *TNFα* were measured by reverse transcriptase–qPCR (RT–qPCR) analysis of EOC2 cells treated with BLZ945 for 3 days found similar trends (Supplementary Fig. S13). Additionally, total RNA was isolated from the OCT-embedded tissue sections and analyzed by RT-qPCR. We found that the mRNA levels of *IL-6* and *TNFα* were the most prominently downregulated in the BLZ945-treated brain specimens of the 4T1-BR5 model (Fig. 6B). A similar downregulation of *IL-6* and *TNFα* was observed in the treated arms of the 231-BR model (Supplementary Fig. S14).

*In vitro* cancer cell invasion was directly impacted by microglial conditioned medium. When BLZ945-conditioned medium was placed in the top chamber of invasion assays 4T1-BR5 cells invaded 40% less than the corresponding conditioned medium from control-treated microglia (Fig. 6C). A similar trend was observed using 231-BR cells, with a 41% reduction in cell invasion in the BLZ945 conditioned microglial medium (Fig. 6C). We tested the effects of IL-6 and TNFα on the invasive ability of 4T1-BR5 cells using a transwell assay. Our results showed that the addition of IL-6, but not TNFα, significantly promoted the invasion of 4T1-BR5 cells *in vitro* (Supplementary Fig. S15). *In vivo*, a prominent tumor cell antiproliferative activity was observed with BLZ945 treatment (Figs. 2F and 4F). Direct application of the BLZ945-microglial conditioned medium, however, had little effect on 4T1-BR5 and 231-BR tumor cell proliferation (Supplementary Fig. S16A-B). Neutralization of IL-6 or TNFα in the 4T1-BR5 or 231-BR cells cultured in microglia-conditioned medium had little effect on inhibiting cell proliferation (Supplementary Fig. S16C,D).

Previous studies have supported the notion that astrocytes have a prometastatic growth effect on brain metastatic cancer cells, including breast cancer [32]. To confirm the contribution of astrocytes to cancer cell proliferation, we used a cancer cell-astrocyte coculture assay with control- or BLZ945-treated microglial conditioned medium [32]. Cancer cell proliferation was not significantly enhanced by coculturing astrocytes with cancer cells. However, the co-culture of BLZ945-microglial conditioned medium with both tumor cells and astrocytes resulted in a 61% and 20% reduction in 4T1-BR5 and 231-BR proliferation, respectively (both *p* < 0.05), compared to the corresponding control-conditioned medium (Fig. 6D and E). Furthermore, the proliferation of cancer cells was increased by the conditioned medium derived from an EOC2 and C8-D1A co-culture compared to the conditioned medium from EOC2 alone (Supplementary Fig. S17). The data, primarily from *in vitro* assays, suggest that CSF-1R signaling in microglia, and possibly brain macrophages, controls not only myeloid proliferation and viability, but also the production of cytokines that modulate the responses of astrocytes and tumor cells in the microenvironment (Fig. 6F).

## Discussion

TNBC is highly aggressive, and approximately 46% of advanced TNBC patients will have a brain metastasis [33]. Given the heterogeneity and instability of brain metastatic tumor cells [34], a potentially promising translational strategy is to target the metastatic microenvironment, which remains incompletely characterized to date. Microglia, the brain’s resident innate immune cells, stimulated brain metastasis development in multiple reports [20, 35–40], although several examples of metastasis suppressive effects have also been published [20, 41]. Microglia aggressively surround and infiltrate brain metastatic lesions, which may include greater numbers of M2-like macrophages compared to their primary breast cancers [42]. The prime microglial-based therapeutics developed to date are small molecule inhibitors and antibodies to the CSF-1R, a critical regulator of the proliferation, differentiation and survival signaling pathway. The goal of the current study was to preclinically credential CSF-1R pathway inhibition for a potential clinical trial in brain metastases derived from TNBC. A monotherapy brain metastasis trial of CSF-1R inhibition could administer the drug with a PFS endpoint, post standard-of-care therapy.

We used BLZ945 in the current set of experiments based on its specificity and potency [28, 43]. A phase I/II trial of BLZ945, alone or in combination with PDR001, was conducted in advanced solid tumor and glioblastoma cohorts (NCT02829723) [44]. The trial was negative but used an intermittent dosing schedule that would not reflect the continuous dosing scheme tested herein. Other small molecule and antibody-based CSF-1R inhibitors have been developed (rev. in [45]). PLX3397, an orally available inhibitor of CSF-1R and KIT, given daily, has received FDA approval for treatment of tenosynovial giant cell tumor [45]. With additional preclinical experiments, any of these inhibitors may be of clinical use.

While literature exists on CSF-1R or myeloid inhibition and brain lesions, the findings vary depending on tumor type, metastasis assay (hematogenous, intracranial implantation), inhibitor, dose, and schedule, with the latter often including pretreatment regimens. Most preclinical brain metastasis experiments in the literature measure the prevention of brain metastasis formation: drug is administered soon after tumor cell injection and continued throughout the experiment, and the number of metastases formed is quantified at necropsy. While scientifically interesting, we are only beginning to design brain metastasis prevention trials and identify endpoints [44]. Using both an immunocompetent 4T1-BR5 model in BALB/c mice and a human 231-BR model in nude mice, we report herein that daily oral administration of BLZ945 significantly reduced both the number and size of brain metastases when administered after metastases had formed (treatment arms), which would be more analogous to standard clinical trial design. This finding, in our experience, is rare. We chose image analysis of brain metastasis number and size as a potentially more specific endpoint of CNS outcomes than overall survival, which can be influenced by lesions in other locations. Brain metastasis trials often use progression free survival (PFS) as an endpoint: formation of a new metastasis, reflected in metastasis number quantification, and increased size of an existing lesion, reflected in metastasis size quantification, are relevant to PFS. The fact that the number of doses administered in each model was distinct, based on the timeline of metastasis development, but were effective nonetheless, strengthens the hypothesis that CSF-1R inhibitor administration may be effective under varying conditions.

Additional variables may be of importance. While weight loss was minimal preclinically, other side effects would require careful investigation. The impact of loss of ~ 25% of microglia on cognition and immunity could be potentially important. Mouse studies under normal physiologic conditions, stroke or neurodegenerative conditions have concluded that short- or long-term microglial depletion did not cause discernable neurologic deficits (rev. in [46]). In some cases, CSF-1R inhibition prevented or ameliorated cognitive deficits: fractionated whole brain radiation therapy caused deficits in hippocampal-dependent memory noted in novel object and matrix distance tests, which was ameliorated by a CSF-1R inhibitor-containing chow [47]. The effect of CSF-1R inhibition on systemic lesions has only been sporadically investigated preclinically [48, 49], and may require combination therapy. Preclinically, glioblastoma and pancreatic cancer preclinical studies suggest CSF-1R inhibition may combine well with checkpoint immunotherapy [50–52], and a study in melanoma suggested a role for myeloid inhibition in ameliorating cognitive adverse effects [52]; clinical trials reported have shown mixed results [45]. Resistance mechanisms to CSF-1R inhibition have been documented [24].

The mechanism of action of CSF-1R inhibition of brain metastases remains complex and incompletely understood. Most reports found that CSF-1R inhibitors reduced the number of both microglia and infiltrating macrophages, the latter including CD206^+^ border-associated macrophages [17, 24, 26]. A switch from an anti-inflammatory to a pro-inflammatory phenotype has been found in myeloid cells treated with CSF-1R inhibitors that limit brain metastasis or intracranial growth [25, 53], and similar findings have been reported in glioblastoma [28]. Herein we noticed that CSF-1R inhibition altered phenotypes of cells other than myeloid cells, including a reduction in astrocyte activation and decreased tumor cell proliferation. Unless the CSF-1R expression of brain and tumor cell types changed between *in vitro* and *in vivo* conditions in a manner that we could not discern, the data suggested that alterations in factors secreted by myeloid cells may be responsible. *In vitro*, BLZ945 decreased microglial expression of cytokines including IL-6 and TNFα. BLZ945 conditioned medium itself did not alter tumor cell proliferation, but combination cultures of BLZ945-treated conditioned medium, astrocytes and tumor cells were antiproliferative. The data, while *in vitro*, support the novel hypothesis that microglia, by their secretion patterns, orchestrate a complex array of tumor-microenvironmental interactions, resulting in metastasis promotion, which can be reduced by CSF-1R inhibition.

## Supplementary Information

Below is the link to the electronic supplementary material.

Supplementary file1 (DOCX 33 KB)

Supplementary file2 (PDF 4875 KB)

Supplementary file3 (XLSX 13 KB)

## Data Availability

No datasets were generated or analysed during the current study.
