# Supplementary material for: A CSF-1R inhibitor both prevents and treats triple-negative breast cancer brain metastases in hematogenous preclinical models
Source: Clin Exp Metastasis. 2025 Aug 4;42(5):45. doi: 10.1007/s10585-025-10366-x (PMC12321670; doi:10.1007/s10585-025-10366-x)
Supplement: Supplementary file 1 — Supplementary file1 (DOCX 33 KB) [file 10585_2025_10366_MOESM1_ESM.docx]

**A CSF-1R inhibitor both prevents and treats triple-negative breast cancer brain metastases in hematogenous preclinical models**

Wei Zhang^1^, Samiur Rahman^1^, Alex M.L. Wu^1^, Kristine Isanogle^2^, Christina Robinson^2^, Dinesh Kumar^1^, Imran Khan^1^, Debbie Wei^1^, [Alexandra S. Zimmer](https://pubmed.ncbi.nlm.nih.gov/?term=Zimmer+AS&cauthor_id=36705597)^1^, Takeo Fujii^1^, Simone Difilippantonio^2^, Stanley Lipkowitz^1^ and Patricia Steeg^1^

^1^Women’s Malignancies Branch, Center for Cancer Research, National Cancer Institute, Bethesda, Maryland.

^2^Animal Research Technical Support, Frederick National Laboratory for Cancer Research, National Cancer Institute, Frederick, Maryland.

Current address for Alex M.L. Wu: Zymeworks Inc., Vancouver BC, Canada; and current address for Alexandra S. Zimmer: Oregon Health and Science University (OHSU), Knight Cancer Institute, Portland, Oregon.

**Corresponding address:** Wei Zhang, Women’s Malignancies Branch, Center for Cancer Research, National Cancer Institute, Building 37, Room 1126, Bethesda, MD 20892. Email: wei.zhang2@nih.gov

**Running Title**: BLZ945 inhibits TNBC brain metastases.

**Keywords**: brain metastasis, triple-negative breast cancer, colony-stimulating factor-1, colony-stimulating factor-1 receptor, BLZ945, microglia, astrocyte, macrophage, tumor microenvironment.

**Supplementary Methods**

**Cell culture**

Brain-tropic 4T1-BR5 and 231-BR cells were cultured in DMEM (Thermo Fisher Scientific) supplemented with 10% FBS (Hyclone), 100 U/ml penicillin/streptomycin (Thermo Fisher Scientific) in 5% CO2 atmosphere at 37 °C as previously described [1]. The mouse brain microglial cell line EOC2 was obtained from the American Type Culture Collection (ATCC # CRL-2467) and cultured in DMEM supplemented with 10% FBS (Thermo Fisher Scientific), 1% GlutaMAX (Gibco), 10 mmol/L HEPES (Thermo Fisher Scientific), 1% penicillin/streptomycin (Thermo Fisher Scientific), and 20% LADMAC-conditioned media. The mouse neuronal astrocyte cell line C8-D1A was also purchased from ATCC (#CRL-2541) and was cultured in DMEM medium containing 10% FBS and 1% penicillin/streptomycin. The mouse brain endothelial cell line (bEnd.3) was cultured in complete classic medium containing serum and Culture Boost (#4Z0-500, Cell Systems). All cell lines were authenticated by DNA fingerprinting through the ATCC and tested negative for Mycoplasma.

**Western blot antibodies**

After blocking with 5% nonfat milk, the following antibodies were used to detect proteins: anti-CSF-1R (#3152, Cell Signaling Technology), anti-phospho-Erk1/2 (#4370, Cell Signaling Technology), anti-Erk1/2(T308) (#4695, Cell Signaling Technology), and anti-β-actin (#A5441, Millipore Sigma).

**Immunofluorescence staining and graphic representation**

Antigen retrieval for deparaffinized tissue sections was performed in acidic citrate buffer for 20 minutes using a steamer. Slides were washed with PBS then incubated in blocking buffer (5% normal donkey serum in PBS) for 30 min at room temperature. Primary antibodies were incubated overnight at 4 °C. After three washes, the secondary antibodies (1:500, Alexa Fluor® antibodies) and DAPI were incubated for 1 h at room temperature. A list of the primary antibodies used can be found in the Supplementary Table 1. The slides were mounted using fluorescence mounting medium (Dako). Images of metastatic lesions and uninvolved areas were captured using a Zeiss Axioskop with a 20× objective. Metastatic lesions were identified through pan-cytokeratin staining or dense DAPI^+^ clusters. Metastatic microenvironment for each metastasis was defined as the region extending 150 µm away from the border of the individual metastatic segment without overlapping into an adjacent metastatic microenvironment. Uninvolved brain regions were defined as any brain area without any visible metastatic lesions within 1 mm. The mean intensity of 4-5 different regions of interest (ROIs) per slide was used for quantification. All immunofluorescence images were acquired and analyzed with the Zeiss Zen software.

**RT-PCR**

Total RNA was extracted using the Qiagen RNeasy Kit (Qiagen) according to the manufacturer's instructions, followed by reverse transcription using the iScript Reverse Transcription Supermix (Bio-Rad). Quantitative real-time PCR (qPCR) was performed using PowerUp SYBR Green Master Mix (Applied Biosystems) on an Applied Biosystems QuantStudio 3 machine. Relative amounts of complementary DNA were calculated using the ΔΔCt method and normalized to GAPDH. The primer sequences are as follows: CSF-1 (F: 5′- GTGTCAGAACACTGTAGCCAC -3′; R: 5′-TCAAAGGCAATCTGGCATGAAG-3′), GM-CSF (F: 5′-AACCTCCTGGATGACATGCCTG-3′; R: 5′-AAATTGCCCCGTAGACCCTGCT-3′), IL-1Ra (F: 5′-TGTGCCTGTCTTGTGCCAAGTC-3′; R: 5′-GCCTTTCTCAGAGCGGATGAAG-3′), IL-6 (F: 5′-TACCACTTCACAAGTCGGAGGC-3′; R: 5′-CTGCAAGTGCATCATCGTTGTTC-3′), TNFα (F: 5′-GGTGCCTATGTCTCAGCCTCTT-3′; R: 5′-GCCATAGAACTGATGAGAGGGAG-3′);

GAPDH (F: 5’-CATCACTGCCACCCAGAAGACTG-3’; R: 5’-ATGCCAGTGAGCTTCCCGTTCAG-3’).

**Microglia conditioned medium**

To prepare the microglia-conditioned medium, the EOC2 cells were cultured until they reached 70% confluence. Then, the cells were treated with either 100nM BLZ945 or DMSO in a medium containing 1% FBS. After 72 hours, the conditioned medium was collected and filtered through a 0.22-μm membrane. It was then applied to mouse cytokine antibody array experiments or cancer cell proliferation assays. To prepare the microglia-astrocyte co-culture conditioned medium, 0.5 × 10⁶ EOC2 cells were seeded with or without 0.5 × 10^6^ C8-D1A in 10-cm plates. The cells were cultured to 70% confluence in EOC2 medium, after which they were switched to DMEM medium containing 1% FBS. After 48 hours, the conditioned medium was collected and filtered through a 0.22-μm membrane. It was then applied to cancer cell proliferation assays.

**Cancer cell–astrocyte coculture experiments and flow cytometry**

A brain metastatic derivative of the triple-negative breast cancer cell line MDA-MB-231, referred to as 231-BR, was transduced with EGFP, as described in a previous study [2]. The 4T1-BR5-mCherry cell line was generated by transfecting the 4T1-BR5 cells with the pCF525-EF1a-Hygro-P2A-mCherry-lenti plasmid (Addgene, Plasmid#115796). 48 hours after transduction, cells were selected with hygromycin B (500 ug/mL, Invitrogen # 10687010) for 2 weeks. Both cell lines showed over 99% positive fluorescence when compared to their parental cells by flow cytometry. Fluorescence-labelled cancer cells and astrocytes were mixed at a ratio of 1:1. The astrocyte cell line C8-D1A was not labelled. 1.0 × 10^5^ cancer cells were seeded with or without 1.0 × 10^5^ astrocytes in 10-cm plates. Cells were cultured in: 1) low serum DMEM medium supplemented with 1% FBS with or without astrocytes; 2) microglia conditioned medium obtained from EOC2 cells treated with 100 nM BLZ945 or DMSO with astrocytes. Cells were cultured for 72 hours prior to analysis. For the cancer cell proliferation assay, the number of mCherry- or EGFP-labelled cancer cells was quantified by flow cytometry (Sony ID7000) in the indicated experiments using the BD Trucount™ Tubes containing a known number of fluorescent beads (BD Biosciences #340334)). Data were calculated and analyzed using the FlowJo version 10 software.

**Hematoxylin and Eosin (H&E) staining and immunohistochemistry (IHC)**

Mouse brain tissue sections were stained with H&E using standard procedures for morphologic evaluation. IHC was performed on 8-μm-thick, 4% paraformaldehyde-fixed, OCT-embedded brain tissue sections [3]. Antigen retrieval for deparaffinized tissue sections was performed in acidic citrate buffer for 20 minutes using a steamer as previously described [3]. After endogenous peroxidase blocking, brain sections were subjected to IHC staining using the Vectastain ABC system (Vector Laboratories) and visualized with DAB (Sigma). Anti-CSF-1R (#3152, Cell Signaling Technology) was used at a dilution of 1:200.

**Transwell invasion assays**

Invasion assays were performed using 8-μm pore Corning Matrigel Invasion Chambers (Corning). For conditioned medium invasion assays, 2.5 ×10^4^ 4T1-BR5 or 231-BR cells in 200 μl conditioned medium from 100 nM BLZ945 or DMSO-treated EOC2 cells were placed in the upper chamber, and 750 µl of DMEM with 5% FBS was placed in the lower chamber. For IL-6 and TNFα invasion assays, 2.5×10^4^ cells in 200 μl of serum-free medium containing 10 ng/ml of IL-6 or TNFα were placed in the upper chamber and 750 µl of DMEM with 5% FBS was added to the lower chamber. Plates were incubated for 12 hours, and then the cells were fixed and stained with Hema 3 Stat Pack (Fisher Healthcare #23-123869). Cells at the bottom of the filters were examined and counted under a microscope at ×200 magnification. Four independent biological replicates were performed for each experiment. Recombinant mouse IL-6 (#406-ML) and TNFα (#410-MT) proteins were purchased from R&D Systems.

**Isolation of bone marrow cells and induction of bone marrow-derived macrophages (BMDM)**

Femurs and tibias were collected from 6–8-week-old female BALB/c mice. Bone marrow was flushed out using a 21G needle attached to a 10 ml syringe filled with cold PBS containing 2% heat-inactivated fetal bovine serum (FBS). The resulting cell suspension was passed through a 70 μm cell strainer to remove clumps, and other unwanted tissue or debris. The bone marrow cells were resuspended in the Iscove's Modified Dulbecco's Medium (IMDM, Millipore Sigma) growth medium at the concentration of 2x10^6^ cells/ml. IMDM was supplemented with 10% FBS and 10 ng/ml M-CSF (R&D Systems, Cat#416-ML). The isolated cells were cultured for five days to generate BMDMs for downstream analyses [4].

**References**

1. Wu AML, et al. (2021) Aging and CNS Myeloid Cell Depletion Attenuate Breast Cancer Brain Metastasis. Clinical Cancer Research 27 15): 4422-34

2. Yoneda T, et al. (2001) A bone-seeking clone exhibits different biological properties from the MDA-MB-231 parental human breast cancer cells and a brain-seeking clone in vivo and in vitro. J Bone Miner Res 16 8): 1486-95

3. Zhang W, et al. (2021) Itraconazole Exerts Its Antitumor Effect in Esophageal Cancer By Suppressing the HER2/AKT Signaling Pathway. Mol Cancer Ther 20 10): 1904-15

4. Ying W, et al. (2013) Investigation of macrophage polarization using bone marrow derived macrophages. J Vis Exp 76)
