# Supplementary material for: A CSF-1R inhibitor both prevents and treats triple-negative breast cancer brain metastases in hematogenous preclinical models
Source: Clin Exp Metastasis. 2025 Aug 4;42(5):45. doi: 10.1007/s10585-025-10366-x (PMC12321670; doi:10.1007/s10585-025-10366-x)
Supplement: Supplementary file 2 — Supplementary file2 (PDF 4875 KB) [file 10585_2025_10366_MOESM2_ESM.pdf]

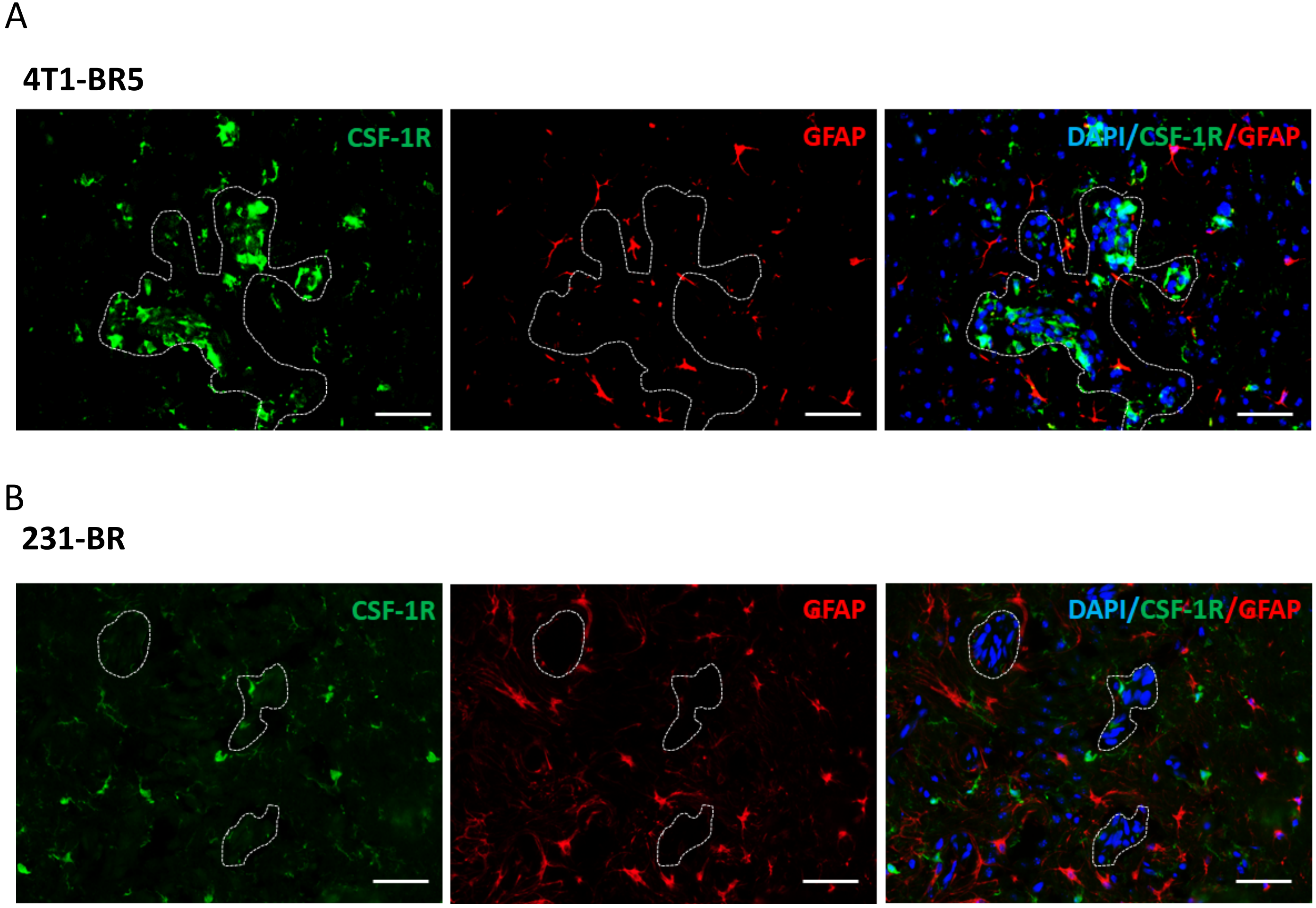

Supplementary Fig. S1: CSF-1R(green) co-staining with GFAP (red) in the metastases and their microenvironments of 4T1-BR5 (A) and 231-BR (B) mouse models of breast cancer brain metastasis. All nuclei are stained with DAPI (blue), and white dotted lines indicate the brain metastases. Scale bar=50 μm for all panels.

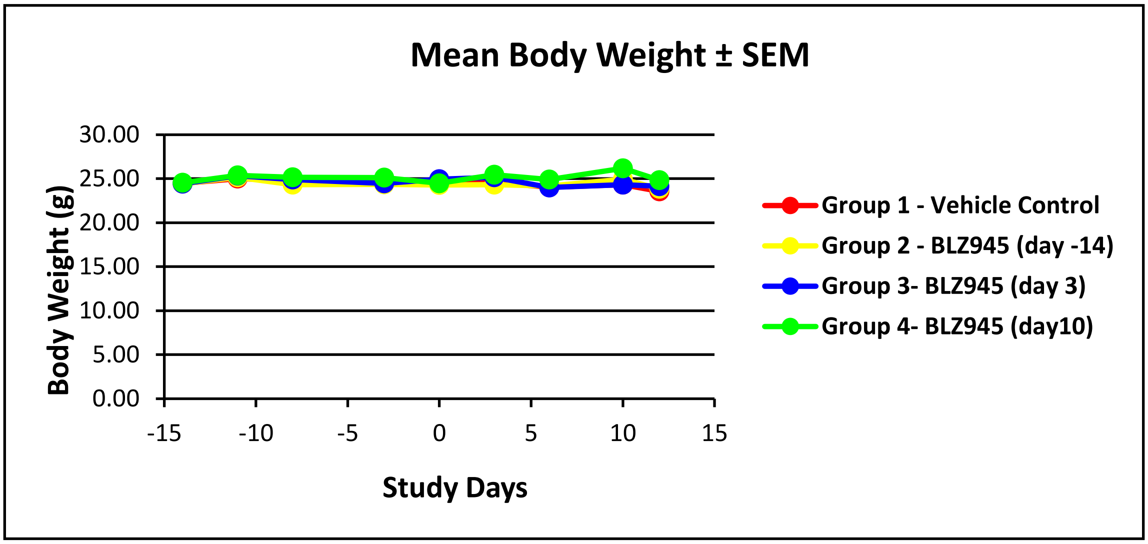

Supplementary Fig. S2: Body weight was monitored in mice in the 4T1-BR5 breast cancer brain metastasis model. No severe toxicity was observed in either control or treatment arms.

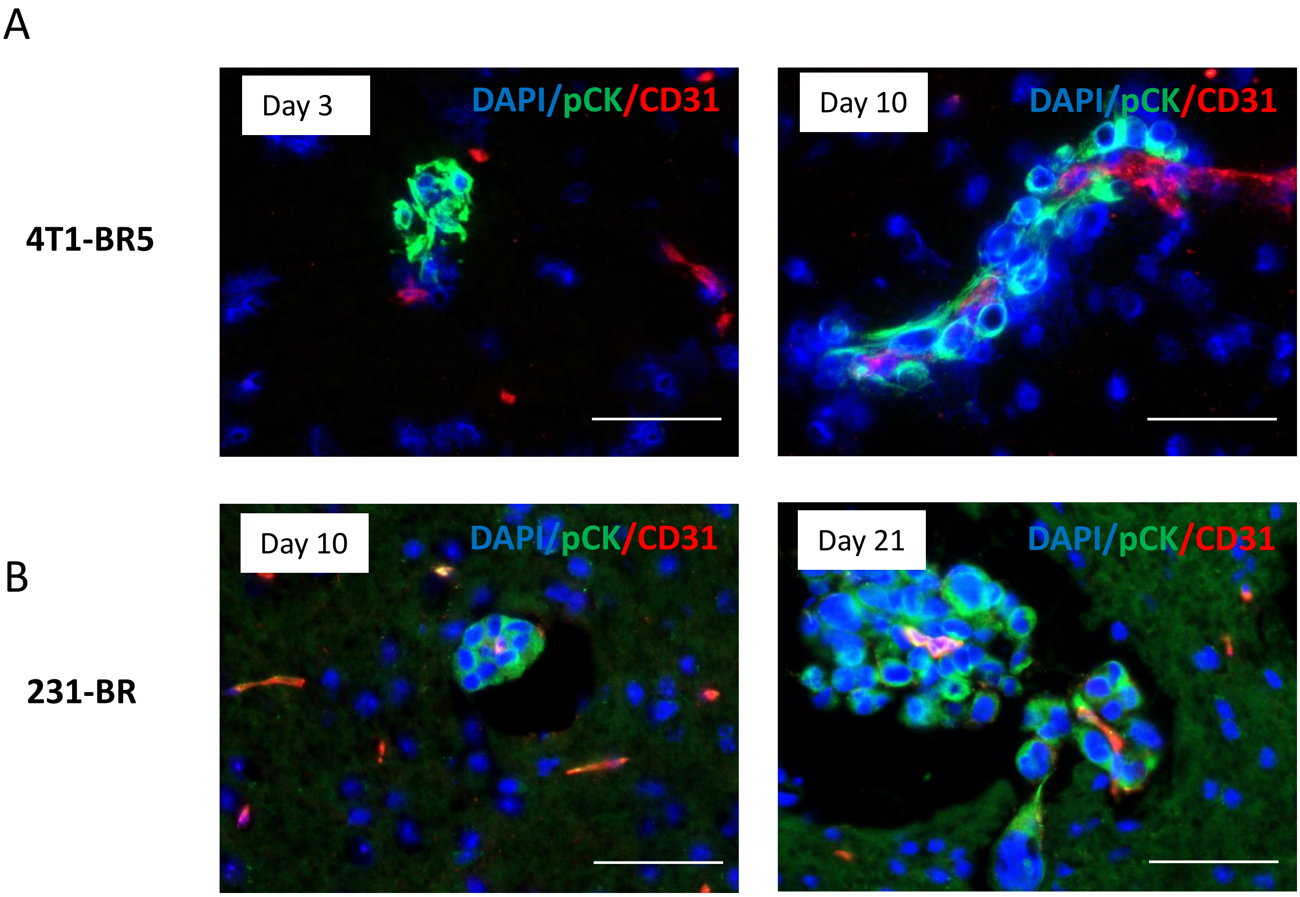

Supplementary Fig. S3: Immunofluorescence staining of pCK (green) and CD31 (red) in the brain micrometastases prior to drug treatment. **A**, Representative images of the 4T1-BR5 model cancer cells in the brain after intracardiac injection at the start of the prevention (day 3) and treatment (day 10) arms. All nuclei are stained with DAPI (blue). Scale bar=50 μm. **B**, Representative images of the 231-BR model cancer cells in the brain after intracardiac injection at the start of the prevention (day 10) and treatment (day 21) arms. All nuclei are stained with DAPI (blue). Scale bar=50 μm.

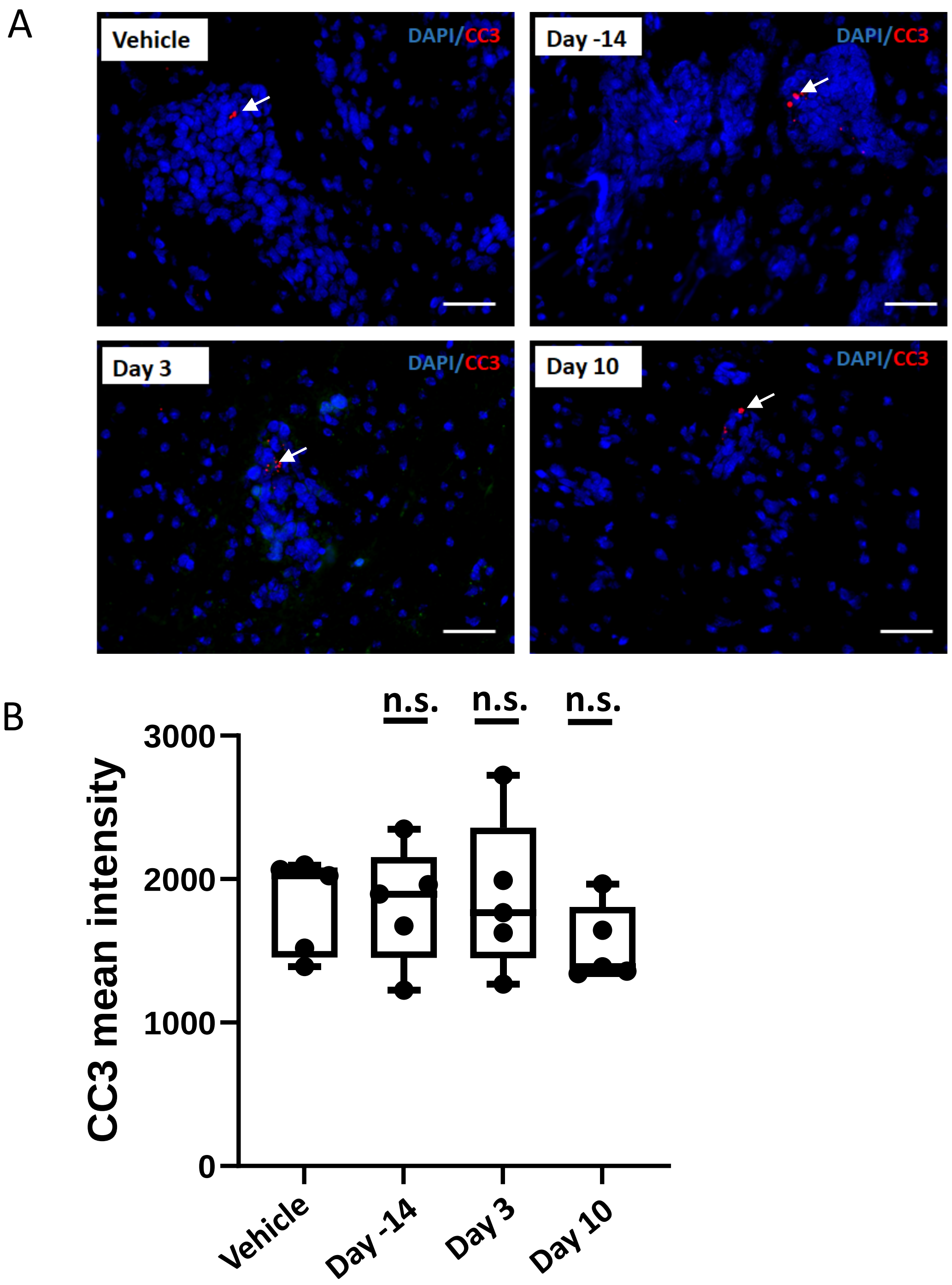

Supplementary Fig. S4: Immunofluorescence staining and quantification of cleaved caspase 3 (CC3, red) in the 4T1-BR5 model at the endpoint in each group. **A**, Apoptosis evaluated by the CC3 staining. All nuclei are stained with DAPI (blue), and arrows indicate the apoptotic cells. Scale bar=50 µm. **B**, Quantification of CC3 fluorescence intensity levels in the metastases from 5 biological replicates. Statistical significance between treatment arms and control was calculated using the Mann–Whitney test (n.s., not significant).

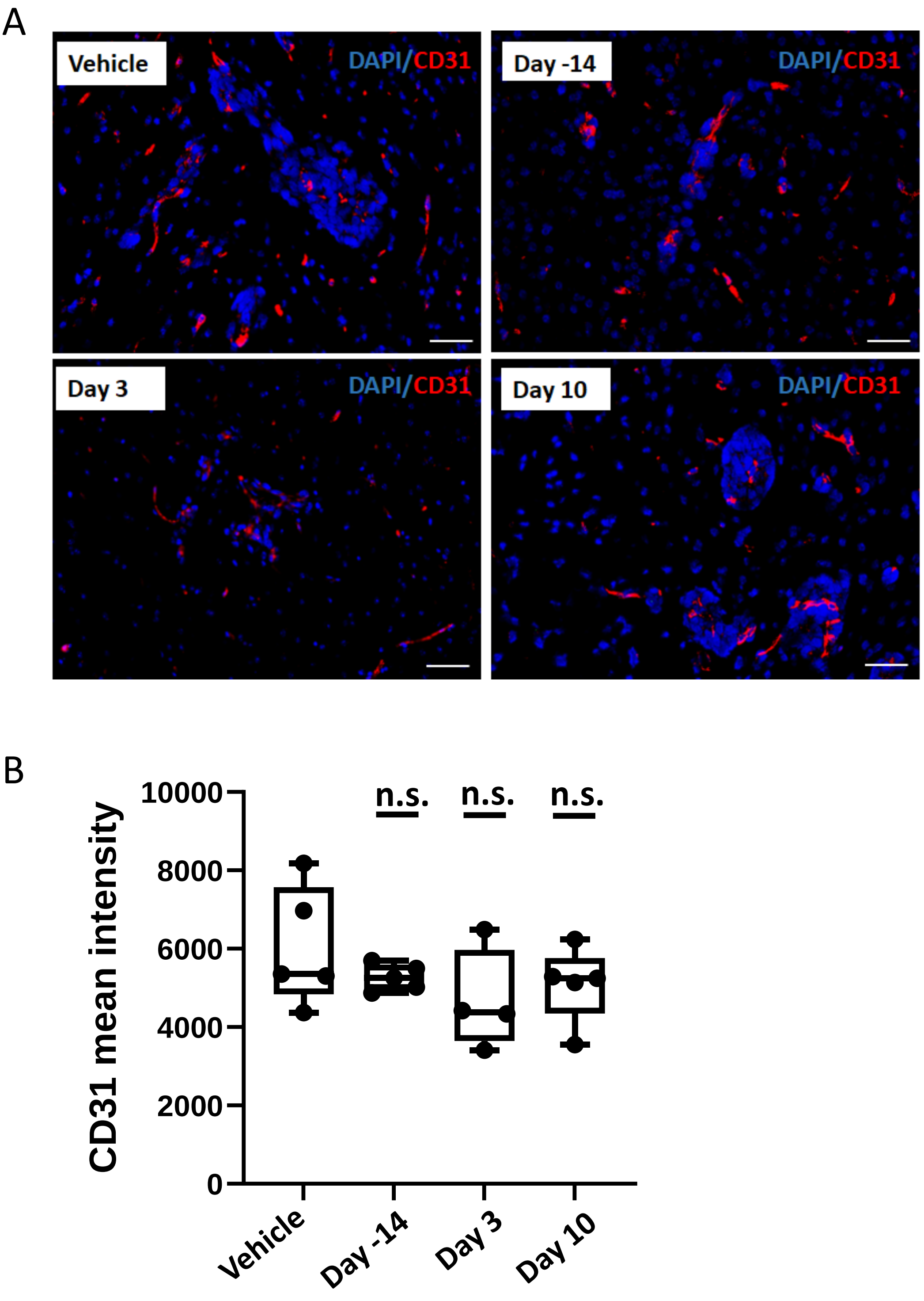

Supplementary Fig. S5: Immunofluorescence staining and quantification of CD31 (red) in the 4T1-BR5 model at the endpoint in each group. **A**, Angiogenesis evaluated by CD31 staining. All nuclei are stained with DAPI (blue). Scale bar=50 μm. **B**, Quantification of CD31 fluorescence intensity levels in the metastases from 5 biological replicates. Statistical significance between treatment arms and control was calculated using the Mann–Whitney test (n.s., not significant).

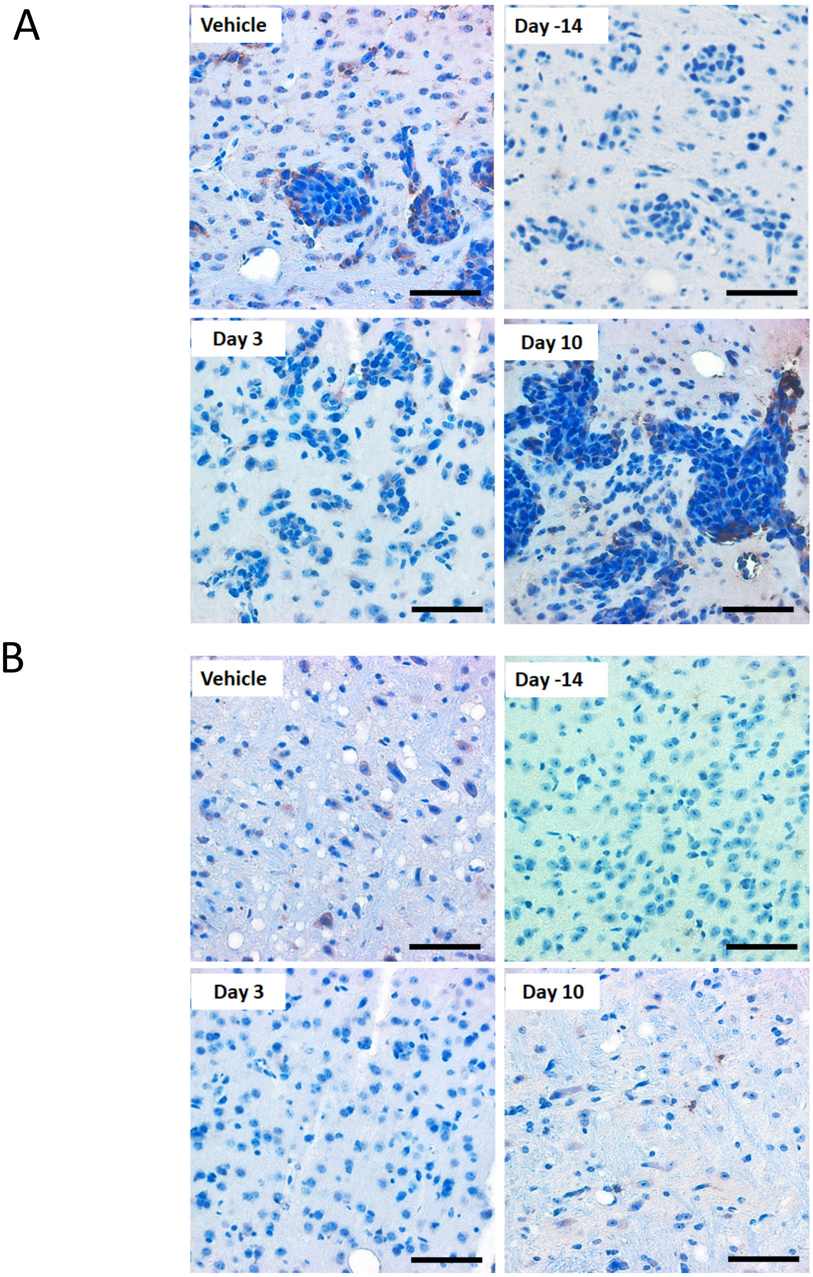

Supplementary Fig. S6: Representative CSF-1R immunohistochemical staining of metastases and their microenvironments (A) and uninvolved brain (B) in control and BLZ945-treated arms at the endpoint. Scale bar=100 μm.

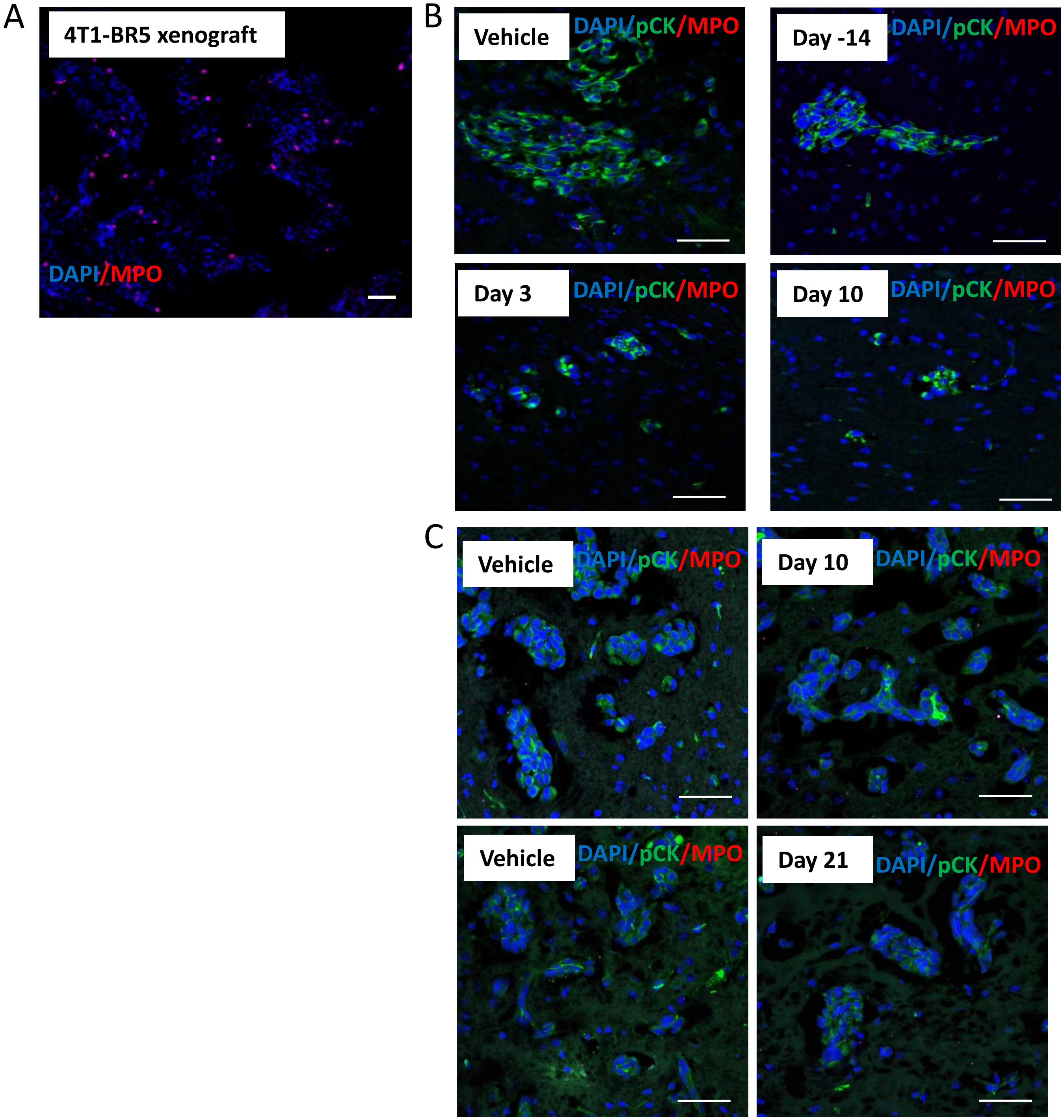

Supplementary Fig. S7: Immunofluorescence staining of MPO (red) in the 4T1-BR5 xenograft (A), the 4T1-BR5 breast cancer brain metastasis model (B), and the 231-BR breast cancer brain metastasis model (C) at the endpoint in each group. Scale bar=50 µm.

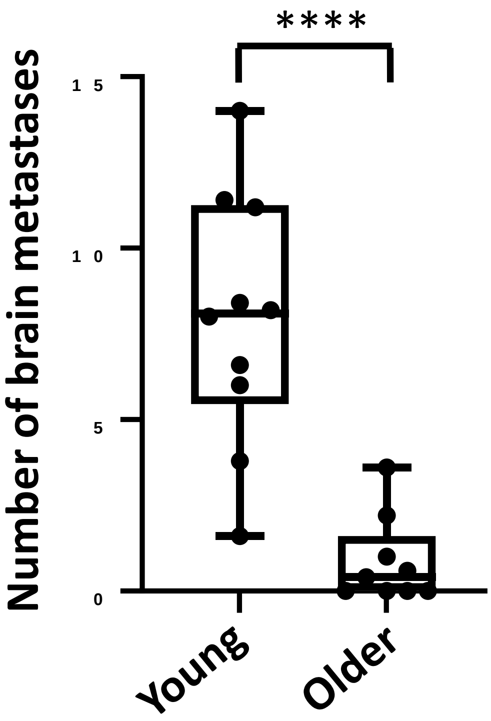

Supplementary Fig. S8: Young mice developed significantly more brain metastases than older mice in the 4T1-BR5 model. Statistical significance was calculated using the Mann–Whitney test (****, $p<0.0001$ older vs young mice; n =9-10).

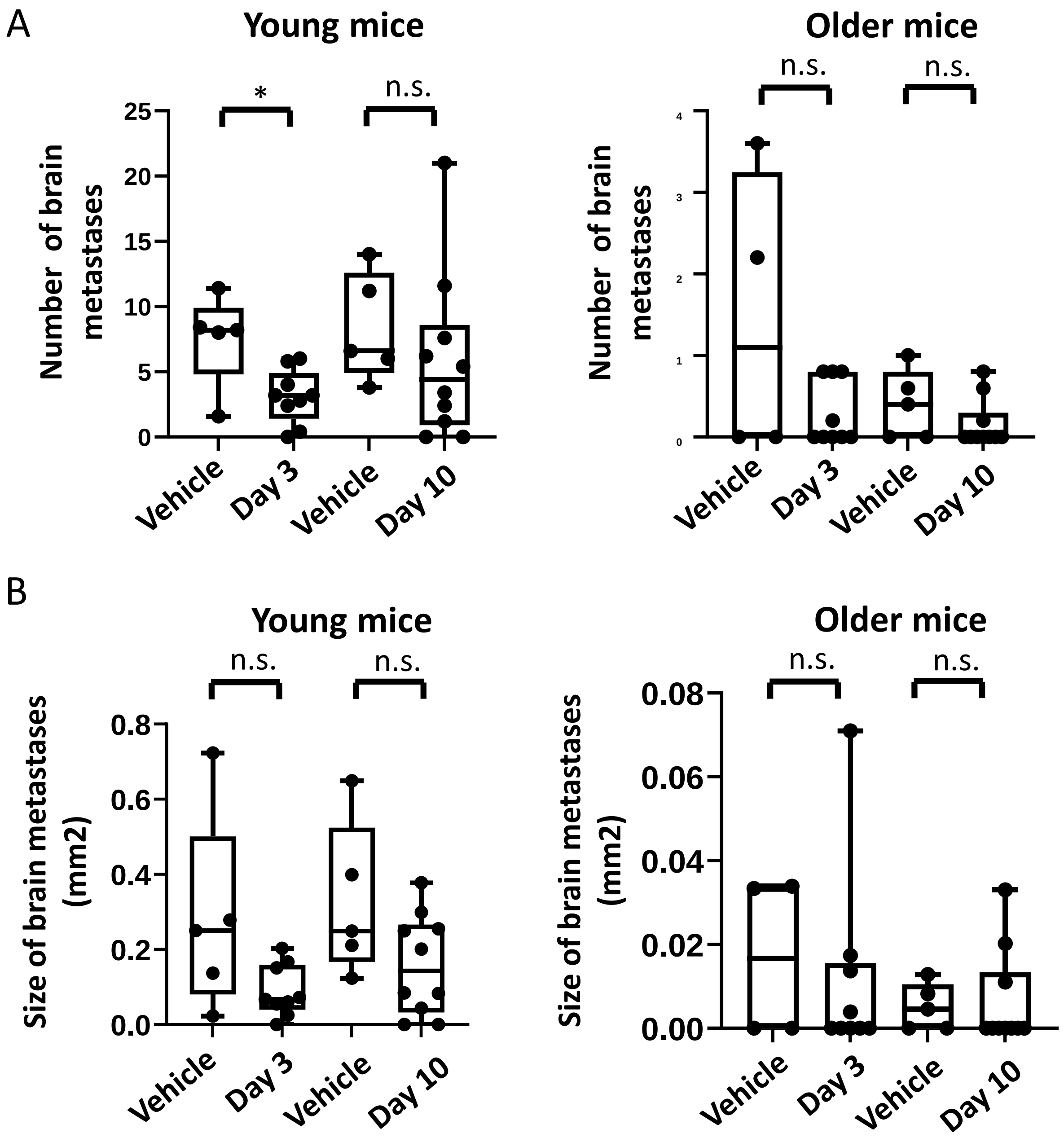

Supplementary Fig. S9: BLZ945 in the prevention and treatment settings of the young mice compared to the older mice. **A**, Number of brain metastases per brain section in young versus older mice treated with vehicle or BLZ945. **B**, Size of brain metastases per animal in young versus older mice treated with vehicle or BLZ945. Young mice were 7 months old and older mice were 18 months old at the time of cancer cell injection. Each dot represents one mouse, and the line designates the group median. n =4-10. *, $p<0.05$ vs vehicle control; n.s., not significant.

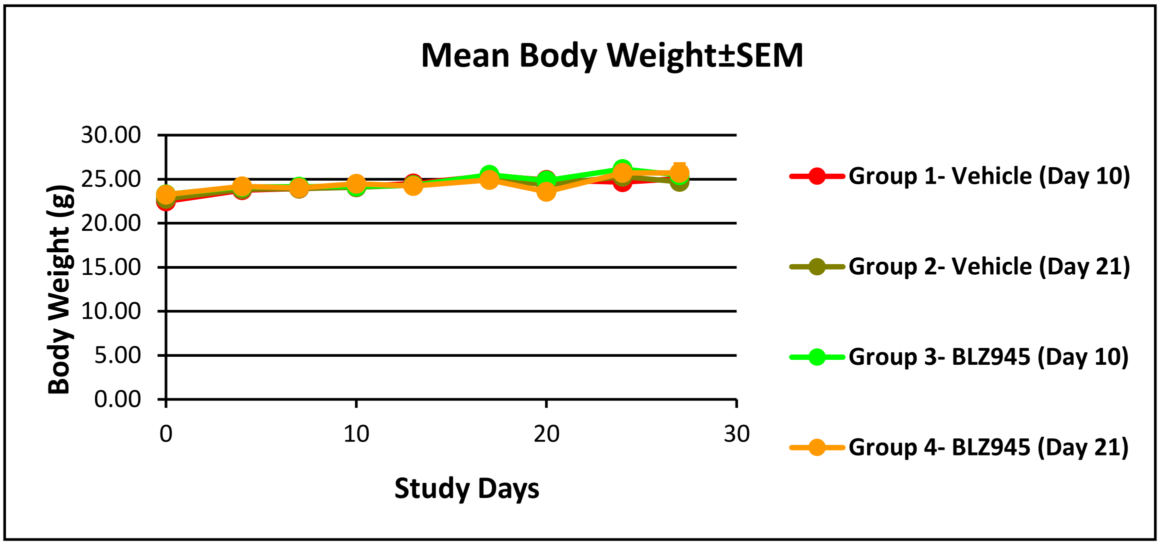

Supplementary Fig. S10: Body weight was monitored in mice during the treatment in the 231-BR breast cancer brain metastasis model. No severe toxicity was observed in either control or treatment arms.

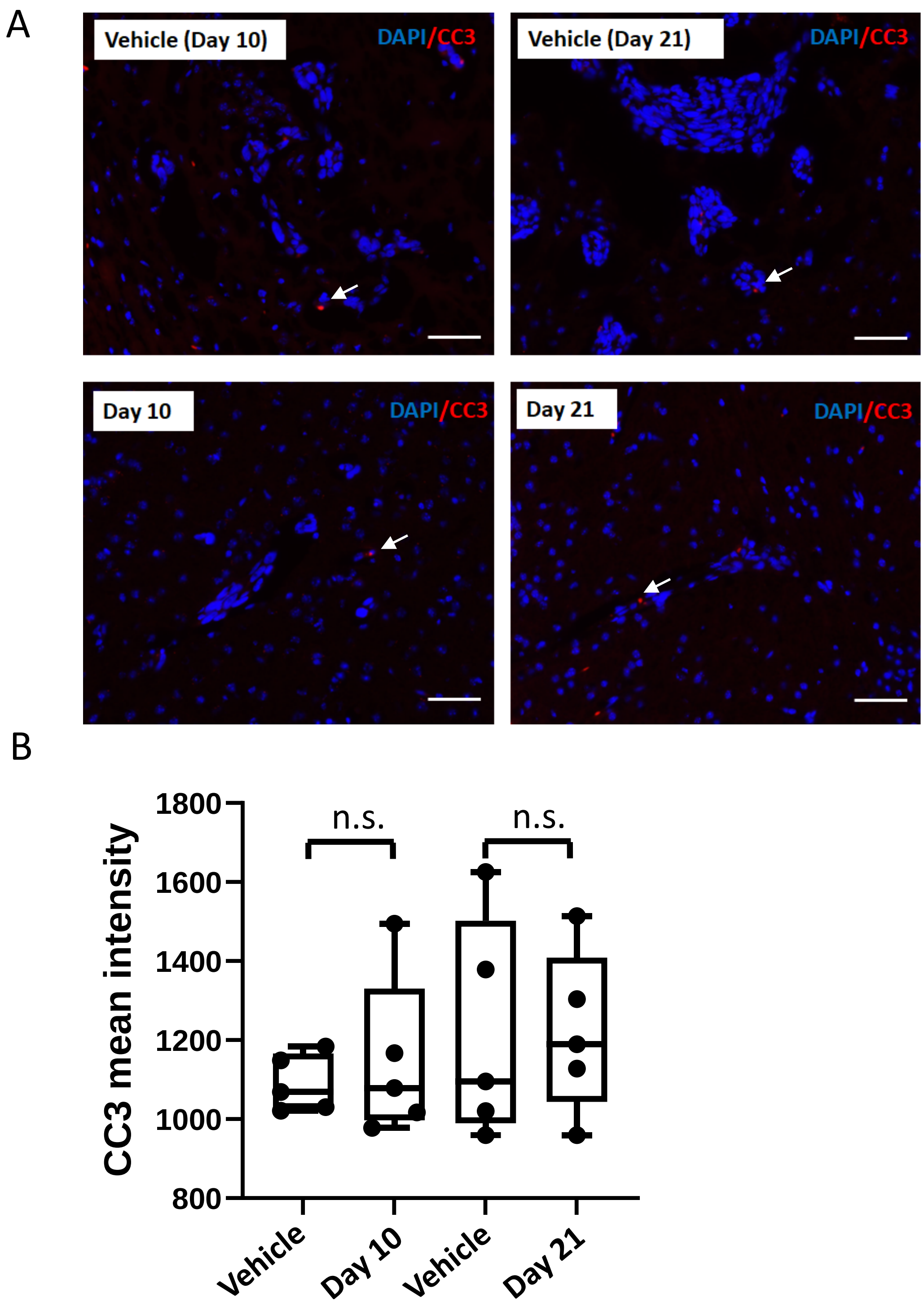

Supplementary Fig. S11: Immunofluorescence staining and quantification of CC3 (red) in the 231-BR model at the endpoint in each group. **A**, Apoptosis evaluated by the CC3 staining. All nuclei are stained with DAPI (blue), and arrows indicate the apoptotic cells. Scale bar=50 μm. **B**, Quantification of CC3 fluorescence intensity levels in the metastases from 5 biological replicates. Statistical significance between treatment arms and control was calculated using the Mann–Whitney test (n.s., not significant).

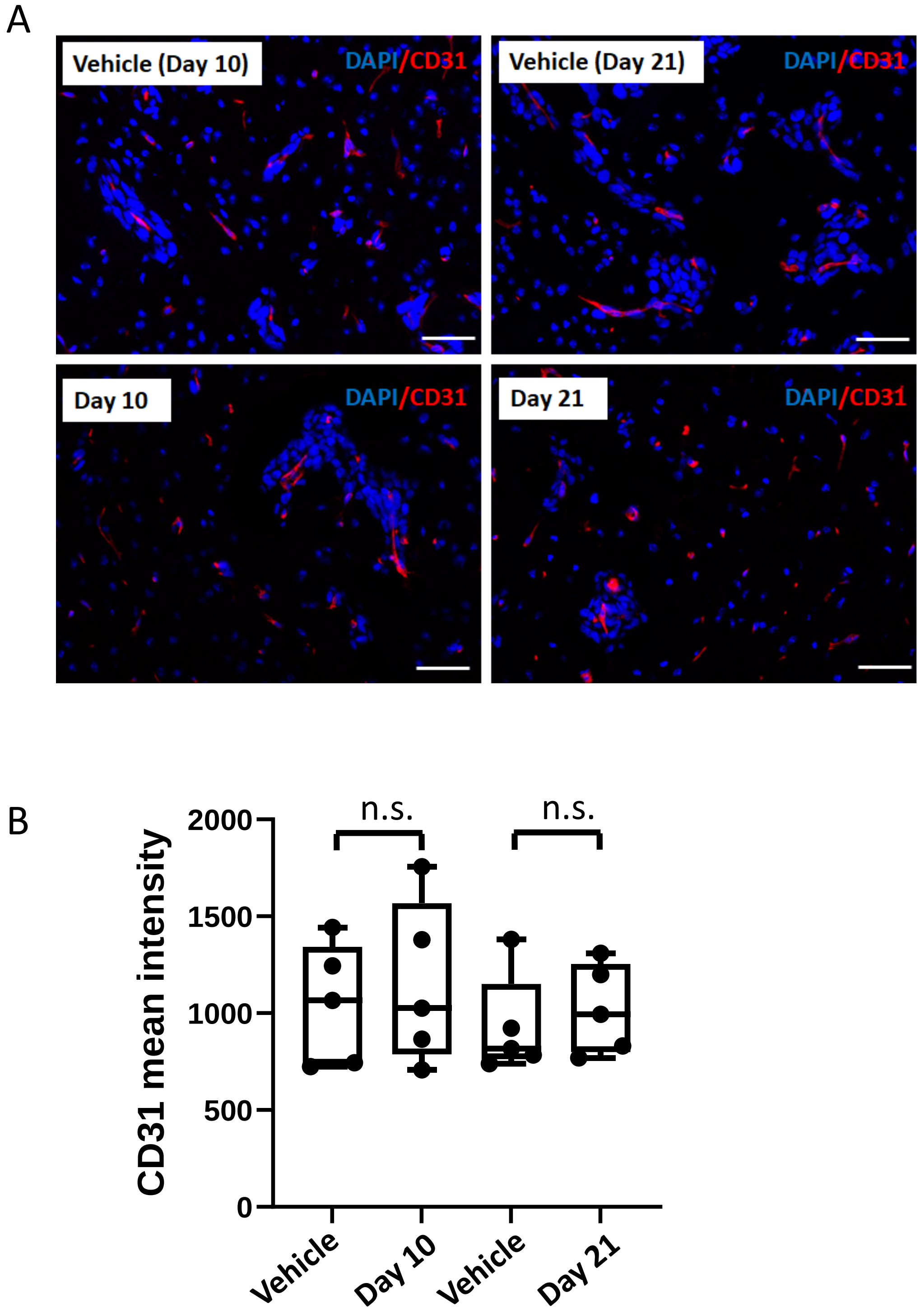

Supplementary Fig. S12: Immunofluorescence staining and quantification of CD31 (red) in the 231-BR model at the endpoint in each group. **A**, Angiogenesis evaluated by CD31 staining. All nuclei are stained with DAPI (blue). Scale bar=50 µm. **B**, Quantification of CD31 fluorescence intensity levels in the metastases from 5 biological replicates. Statistical significance between treatment arms and control was calculated using the Mann–Whitney test (n.s., not significant).

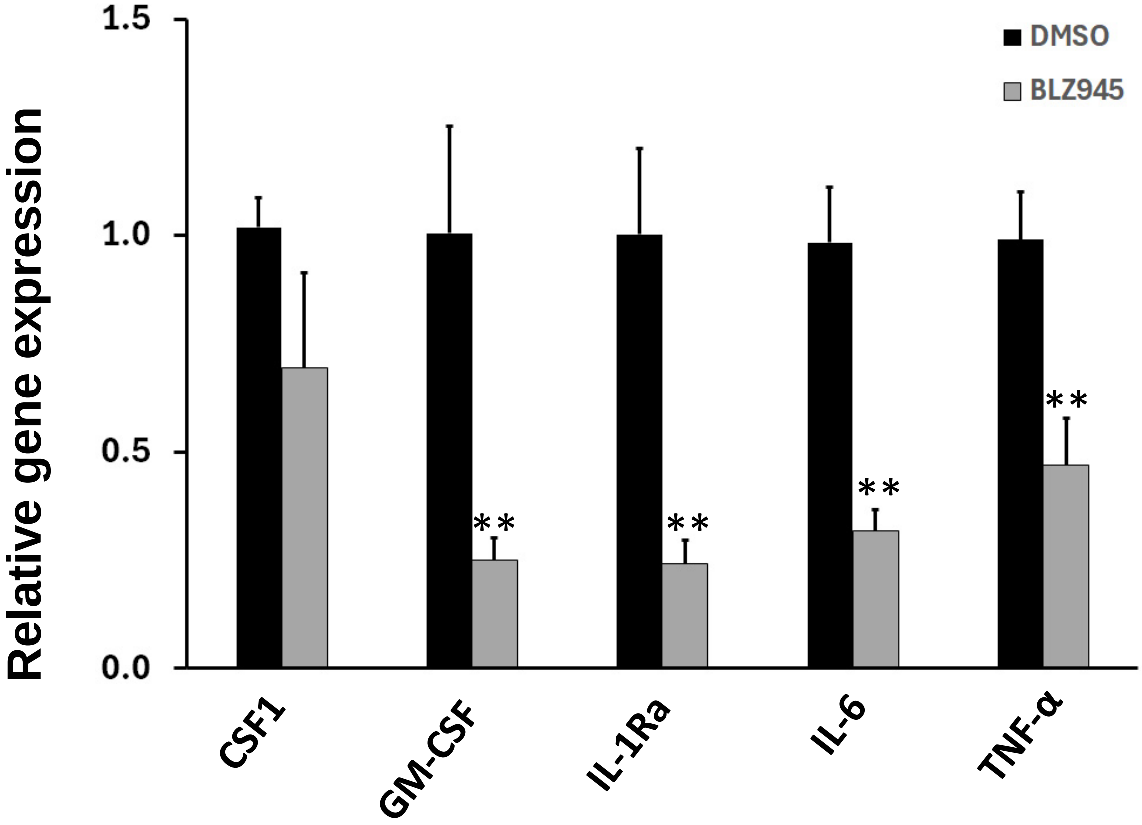

Supplementary Fig. S13: RT-PCR validation of microglial cytokine production. Values represent the mean fold change ±SD for three experiments relative to GAPDH. Black bars, DMSO; gray bars, BLZ945. **, $p<0.01$ vs DMSO control by the Mann–Whitney test.

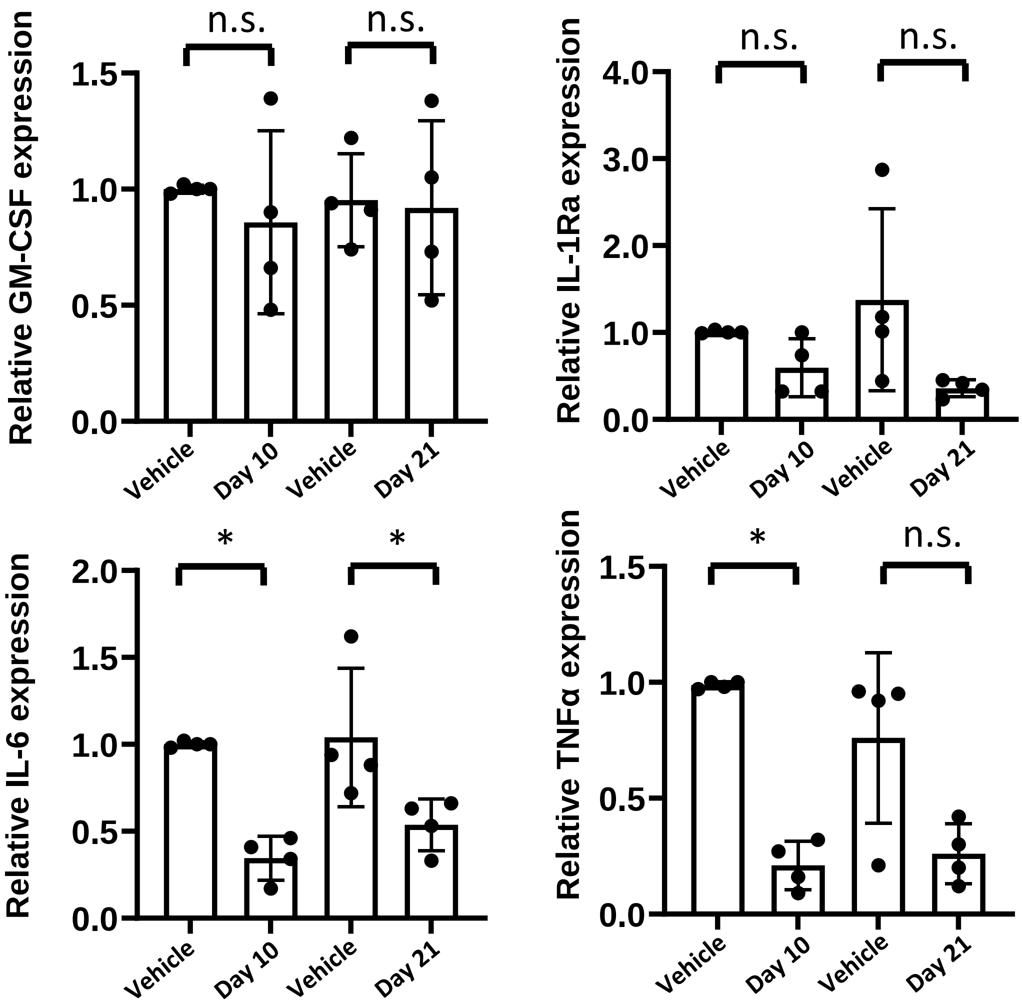

Supplementary Fig. S14: RT-PCR validation of cytokine expression in the brain of the 231-BR breast cancer brain metastasis model. Values represent the fold change ±SD relative to vehicle. Each dot represents one mouse, and the line designates the group median. Statistical significance between the treatment arms and the control groups was calculated using the Mann–Whitney test (*, $p<0.05$; n.s., not significant).

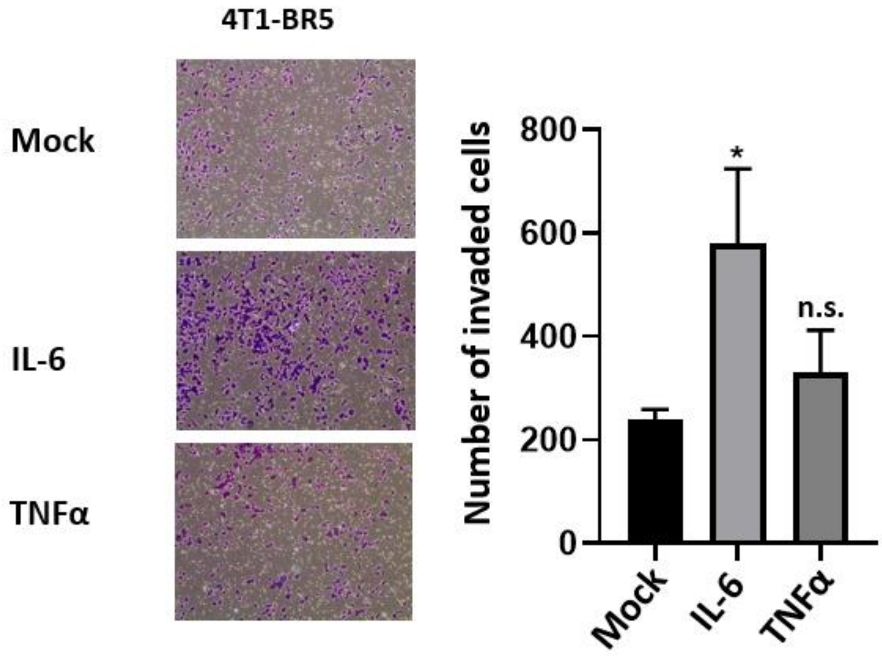

Supplementary Fig. S15 : IL-6 significantly increased 4T1-BR5 cell invasion. 4T1-BR5 cells were allowed to invade through Matrigel for 12 h in the presence or absence of recombinant mouse IL-6 (10 ng/ml). Invaded cells were counted using ImageJ software. $n \geq 3$ independent experiments. *, $p<0.05$ IgG control; n.s., not significant by the Mann–Whitney test.

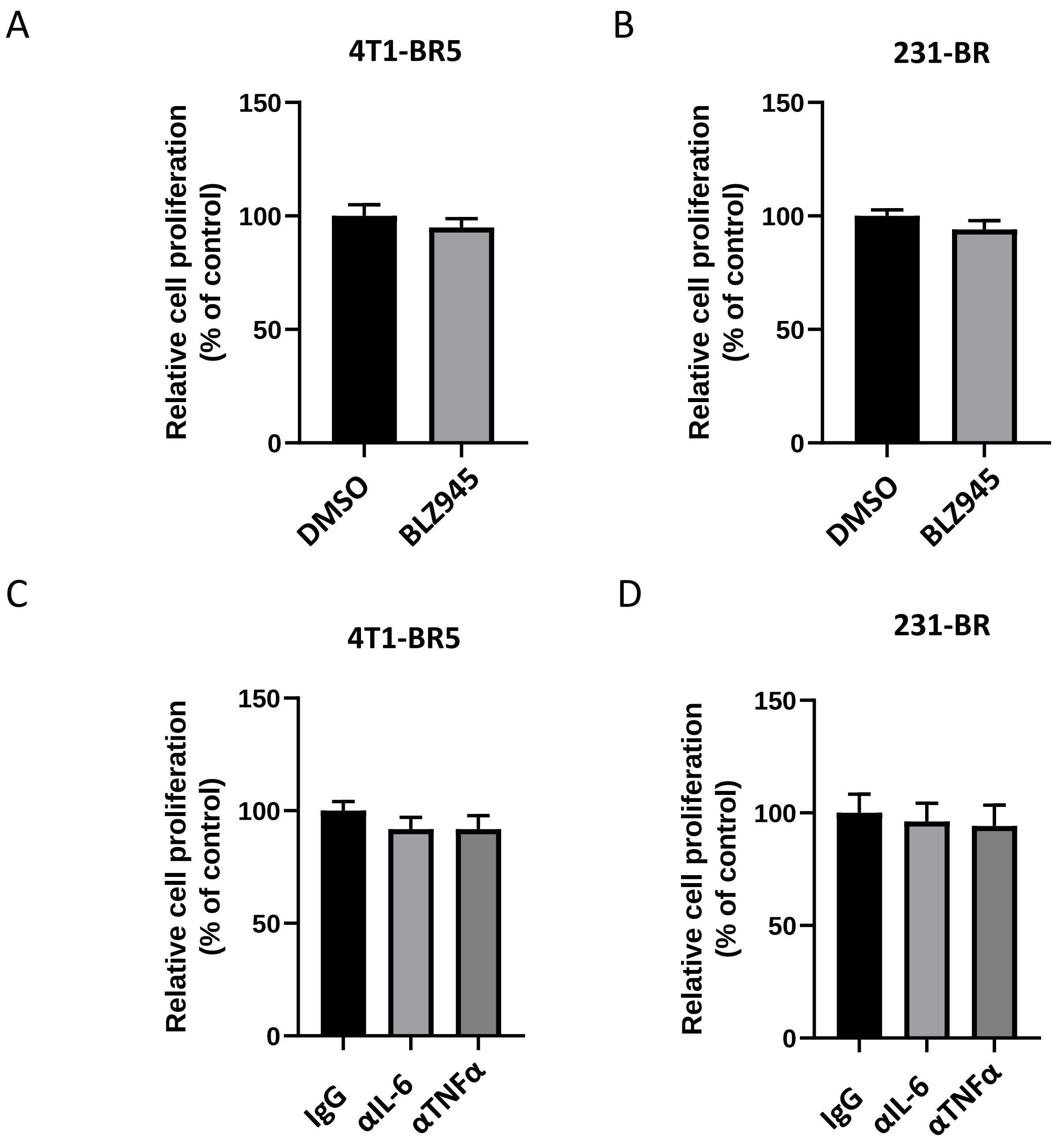

Supplementary Fig. S16: EOC2-derived conditioned medium had no direct effect on cancer cell proliferation. **A-B**, 4T1-BR5 and 231-BR were exposed to EOC2 conditioned medium for 72 hours. Conditioned medium was collected from EOC2 cells treated with either DMSO or 100 nM BLZ945 for 72 hours. **C-D**, 4T1-BR5 and 231-BR cells were grown in EOC2 conditioned medium supplemented with 10 µg/ml isotype IgG control, IL-6 neutralizing antibody, or TNFα neutralizing antibody for 72 hours. Cell proliferation was measured by the alamarBlue assay. Values represent the mean fold change ±SD relative to control conditioned medium (*n*=5). No statistical significance was found using the using the Mann–Whitney test.

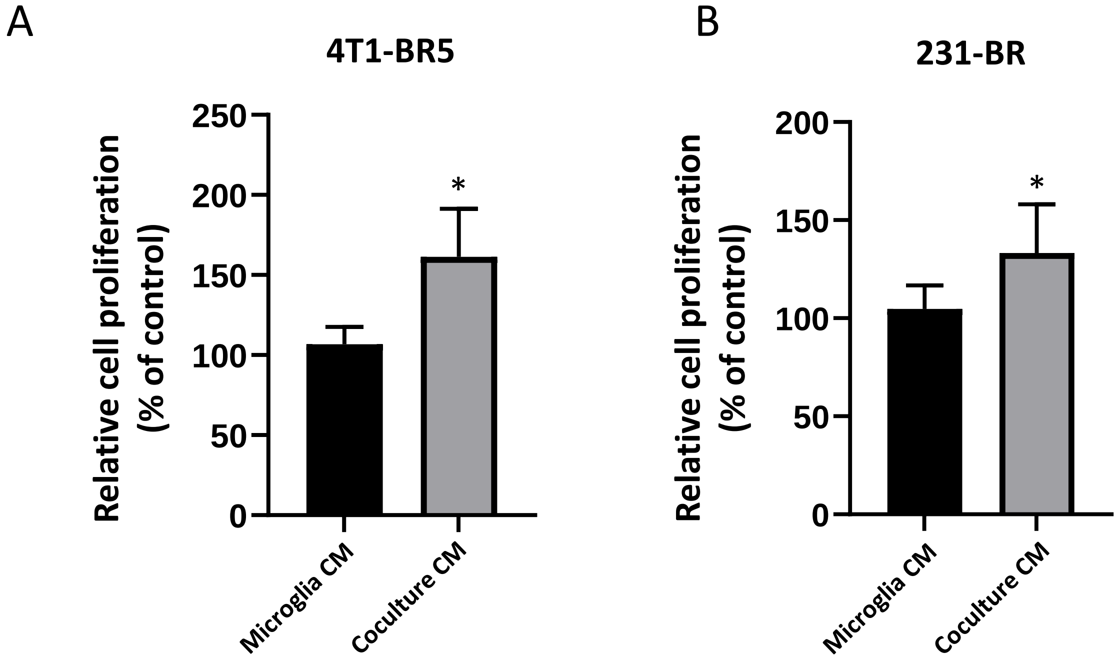

Supplementary Fig. S17: Conditioned medium derived from a microglia and astrocyte coculture promotes cancer cell proliferation. 4T1-BR5 (A) and 231-BR (B) cells were treated with conditioned medium from EOC2 alone or from an EOC2 and C8-D1A coculture for 72 hours. Cell proliferation was measured by the alamarBlue assay. Values represent the mean fold change ±SD relative to control conditioned medium (*n*=6). *, $p<0.05$ by the Mann–Whitney test.
